# Ultrasonic Control of Polymer-Capped Plasmonic Molecules

**DOI:** 10.1021/acsnano.4c10912

**Published:** 2024-10-31

**Authors:** Yingying Cai, Swagato Sarkar, Yuwen Peng, Tobias A. F. König, Philipp Vana

**Affiliations:** † Institut für Physikalische Chemie, Georg-August-Universität Göttingen, Tammannstrasse 6, 37077 Göttingen, Germany; ‡ Leibniz-Institut für Polymerforschung Dresden e.V., Hohe Straße 6, 01069 Dresden, Germany; § Center for Advancing Electronics Dresden (cfaed), Technische Universität Dresden, Helmholtzstraße 18, 01069 Dresden, Germany; ∥ Faculty of Chemistry and Food Chemistry, Technische Universität Dresden, Bergstraße 66, 01069 Dresden, Germany

**Keywords:** self-assembly, plasmonic molecules, supracolloidal assemblies, nanostructures, ultrasound-responsiveness, polymer, hydrogen bond

## Abstract

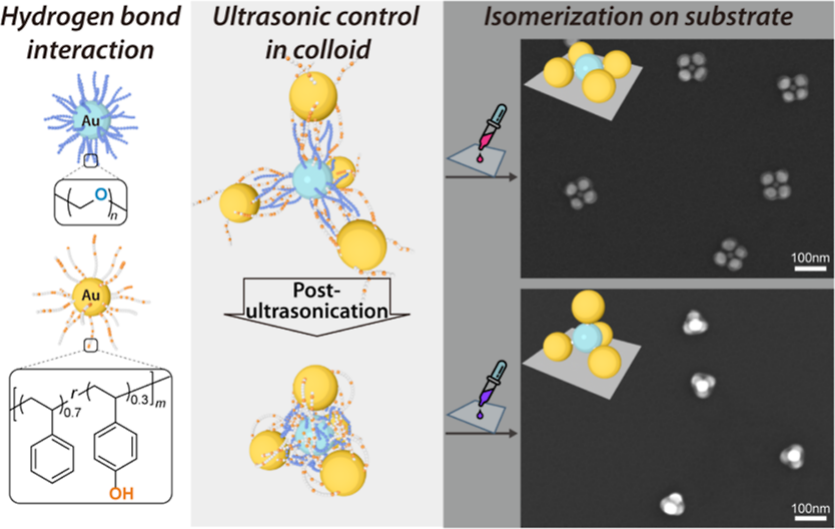

Plasmonic molecules (PMs) composed of polymer-capped nanoparticles represent an emerging material class with precise optical functionalities. However, achieving controlled structural changes in metallic nanoparticle aggregation at the nanoscale, similar to the modification of atomic structures, remains challenging. This study demonstrates the 2D/3D isomerization of such plasmonic molecules induced by a controlled ultrasound process. We used two types of gold nanoparticles, each functionalized with hydrogen bonding (HB) donor or acceptor polymers, to self-assemble into different AB_*N*_-type complexes via interparticle polymer bundles acting as molecular bonds. Post-ultrasonication treatment significantly shortens these bonds from approximately 14 to 2 nm by enhancing HB cross-linking within the bundles. This drastic change in the bond length increases the stiffness of the resulting clusters, facilitating the transition from 2D to 3D configurations in 100% yield during drop-casting onto substrates. Our results advance the precise control of PMs’ nanoarchitectures and provide insights for their broad applications in sensing, optoelectronics, and metamaterials.

## Introduction

Plasmonic nanoparticles (NPs), e.g., Au and Ag, exhibit localized surface plasmon resonance when their conduction electrons oscillate in resonance with incident light.^1^ Assembling these NPs into clusters introduces new collective optical properties that are hybridized from the plasmonic coupling between its primary building blocks.^2^ Controlling plasmonic assemblies in terms of particle geometry, materials, and spatial arrangement provides a potent means to manipulate light–matter interactions;^1,3−5^ these assemblies thus hold significant potential in various fields, such as sensing,^6,7^ optoelectronics,^8,9^ and metamaterials.^10,11^

Plasmonic molecules (PMs) are a class of plasmonic assemblies that mimic molecular structures.^1,10,12^ Their plasmon modes follow symmetry rules similar to those of real molecules, where atomic orbitals hybridize to form molecular orbitals, thus offering predictable optical behaviors.^1,5,13^ For molecules, it is well known that different isomers (e.g., tetrahedral vs square planar for AB_4_-molecules) exhibit distinctly different hybridizations. Understanding and controlling the structural isomerization of PMs can lead to advancements in designing optical devices with tunable properties.^13−15^ Despite these advancements, achieving such 2D/3D isomerization of PMs remains a significant challenge. Although various assembly methods have been developed, realizing different isomers still necessitates a complex redesign of assembly pathways. The lithographic method provides convenient access to programming structures larger than 10 nm but is generally restricted to 2D patterns.^16,17^ DNA-scaffolding, while capable of customizing diverse architectures, requires dedicated design and fabrication efforts for each structure.^18,19^ Solvent-assisted templating can produce 3D structures in the form of larger superlattices^7,20^ but tends to yield 2D formations for much smaller clusters due to capillary forces.^11,21^ Once these nanostructures are completed, their spatial arrangement is fixed and is rarely able to be rearranged.

In recent years, colloidal self-assembly has made significant strides in fabricating nanostructures with high structural fidelity and efficiency.^3,15,22^ Structures such as chain-like,^23,24^ molecular-like,^25−27^ and core-satellite^28−30^ configurations have been achieved through sophisticated balancing of close-range attractive forces to bond NPs and long-range repulsive forces to confine the assembly degree.^31,32^ In this context, synthetic polymer-capped NPs have shown great potential. The polymer shells not only facilitate precise control over interparticle interactions through monomer formulation, e.g., via electrostatic interactions,^23^ acid–base neutralization,^25,27^ and hydrogen bonding (HB),^26,30^ but also provide necessary steric hindrance that aids in controlling the assembly’s structure.^27,33^ For instance, our recent work has demonstrated the efficacy of using a pair of polymer-capped AuNPs, each with HB-donor and HB-acceptor moieties, to construct AB_*N*_-type PMs with accurate control over the coordination number *N*.^26^

Moreover, polymer engineering is versatile in tuning mechanical properties through architectural design elements such as composition, chain length, and cross-linking.^34,35^ Functional polymers can respond to external stimuli (e.g., temperature,^36,37^ pH,^38,39^ light,^40,41^ and ultrasound^42−46^), creating design space for post-assembly modulation of the constructed nanoarchitecture.

Here, we present an innovative approach for 2D/3D isomerization of PMs by post-engineering HB polymer linkages. Using HB-interacting polymer-functionalized AuNPs, we assembled AB_*N*_-type PMs in the colloidal form. The interconnecting polymer bundles (i.e., the PM bonds) formed between A- and B-type NPs somewhat mimic σ-bond formation from atomic orbital hybridization. Post-ultrasonication significantly enhances HB cross-linking within the polymer bundles by facilitating the ejection of solvent molecules. This stimulus transitions PM bonds from soft to rigid efficiently, which further results in distinct 2D or 3D configurations of PMs during drop-casting on the substrate. Notably, the structural stiffness of PMs directly correlates with the interparticle distance between A- and B-NPs, analogous to molecular bonds where shorter lengths correspond to higher energies. We have studied several PM isomers, including AB_4_, AB_6_, AB_8_, and AB_12_, and have gained insights into their transformations in both colloidal and solid states. This study combines experimental observations with optical simulations to explain the mechanisms driving these transitions, setting the foundation for a deeper discussion of the implications and applications of our findings.

## Results and Discussion

### Isomerization of PMs by Post-ultrasonication

**Figure 1:**
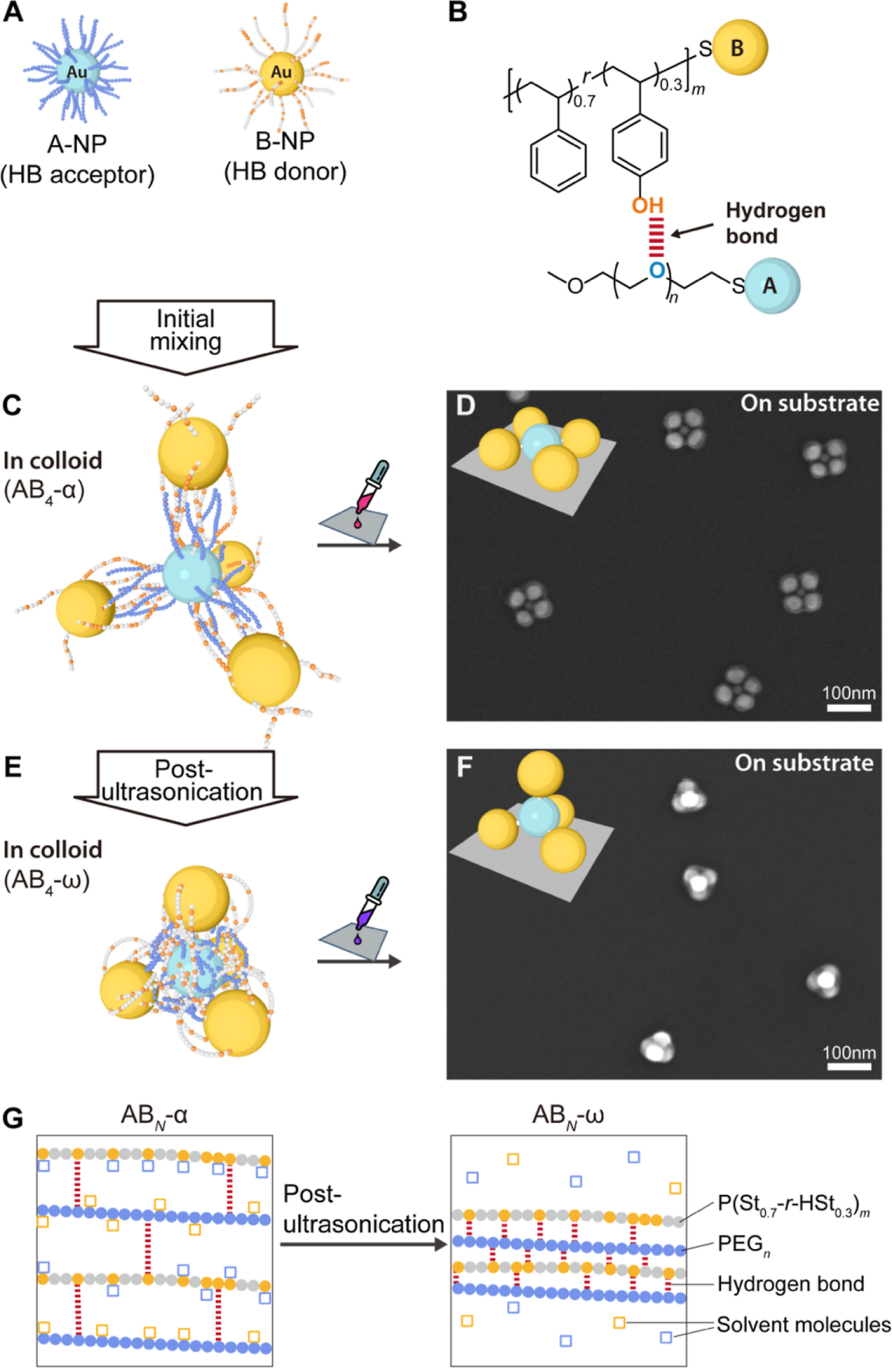
Schematic of self-assembly and isomerization of PMs via HB interaction. (A) Building blocks: PEG_*n*_ (HB acceptor) and P(St_0.7_-*r*-HSt_0.3_)_*m*_ (HB donor) grafted onto AuNPs. *m* and *n* refer to the number of repeating units. (B) HB-donor and -acceptor polymer design and their interaction when grafted onto the AuNPs. (C) Mixing A- and B-NPs forms colloidal PMs (AB_4_-α) by forming polymer bundles (PM bonds). (D) Scanning electron microscopy (SEM) image: AB_4_-α colloid transfers to a 2D square planar on a substrate. (E) Upon post-ultrasonication, AB_4_-α changes to the AB_4_-ω state in the colloid. Polymer bundles shrink, and the polymer chains entangle tightly. This results in a decrease in the PM bond length and an increase in the structural stiffness. (F) SEM image: drop-casting AB_4_-ω achieves a tetrahedral isomer due to increased stiffness. (G) Ultrasonication ejects solvent molecules, forming new HB linkages and transitioning from AB_*N*_-α to AB_*N*_-ω.

As illustrated in Figure 1B, a thiol-terminated random copolymer of styrene and hydroxystyrene (P(St_0.7_-*r*-HSt_0.3_)_*m*_) is utilized for its HB donating ability via the phenol group. This copolymer is synthesized via reversible addition–fragmentation chain-transfer (RAFT) polymerization, followed by hydrolyzation and reduction steps (Figure S1). Commercial thiol-terminated poly(ethylene glycol) methyl ether (PEG_*n*_) of varying chain length is used for its ether group as HB-acceptors (*m* and *n* are both repeating units). Cetyltrimethylammonium chloride (CTAC)-stabilized AuNPs are synthesized by seed-mediated growth methods with diameters of 22, 28, 30, and 34 nm. These two types of polymers are grafted onto the surface of AuNPs via a ligand exchange process, forming a covalent thiol–gold bond (Figures S14–18, and Table S1). The high Au–S binding strength (170 kJ/mol)^47^ ensures robust anchoring of the polymer brushes on the Au surface (Figure S23), preventing detachment during ultrasonication. HB-acceptor NPs (i.e., A-NPs, with a PEG_*n*_ shell) and HB-donor NPs (i.e., B-NPs, carrying a P(St_0.7_-*r*-HSt_0.3_)_*m*_ shell) were then prepared as stock colloids serving as the building blocks for the subsequent PM assembly process (Figure 1A).

The self-assembly process is performed by mixing the A- and B-NPs in predetermined ratios under optimized conditions.^26^ Samples are then taken after different conditions (ultrasonication and incubation time) for UV–vis spectroscopy and dynamic light scattering (DLS) analysis to monitor changes in the colloidal states. After drop-casting onto a substrate, SEM measurements are conducted to determine the assemblies’ geometry in their dried state. A binary solvent mixture of tetrahydrofuran (THF, a moderate HB donor^48,49^) and chloroform (CHCl_3_, a weak HB acceptor^48,49^) is used to provide an appropriate level of solvation for each HB moiety.

**Figure 2:**
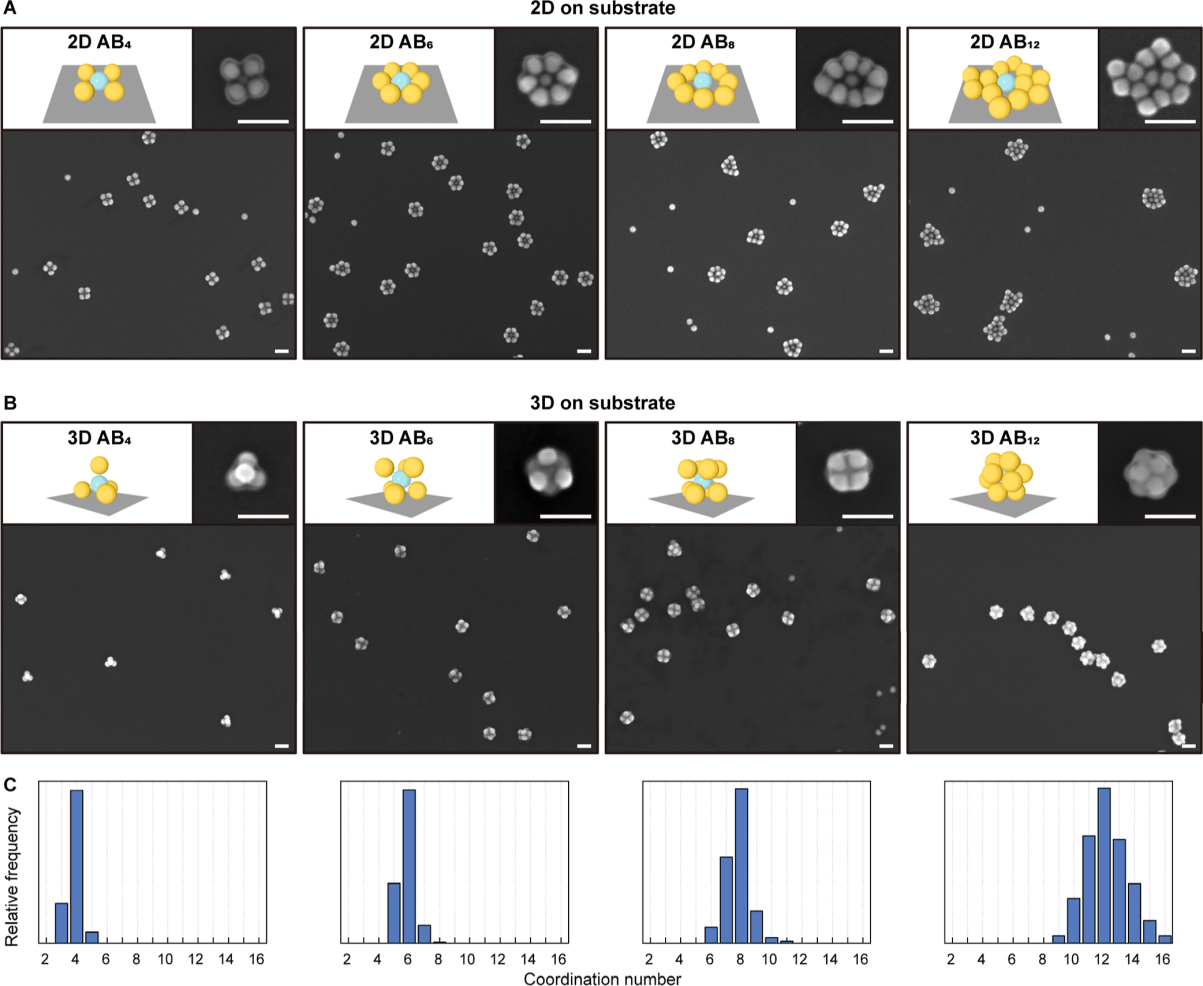
PMs with different coordination numbers and configurations on the substrate. (A) SEM images of AB_*N*_ PMs in 2D configurations for *N* = 4, 6, 8, and 12 prepared by drop-casting AB_*N*_-α colloid on a silicon wafer. Insert illustrations show the spatial arrangement on the substrate schematically. The A-NPs used are PEG_136_-grafted 22 nm A-NPs for *N* = 4, PEG_227_-grafted 28 nm A-NPs for *N* = 6 and 8, and PEG_454_-grafted 30 nm A-NPs for *N* = 12. All samples utilize 34 nm B-NPs grafted with P(St_0.7_-*r*-HSt_0.3_)_420_. (B) SEM images of AB_*N*_ PMs in 3D configurations, prepared using AB_*N*_-ω colloid, which underwent post-ultrasonication from the corresponding AB_*N*_-α colloid. Inset illustrations show the typical schematic spatial arrangement on the substrate. (C) Statistical distribution of *N* values for AB_4_, AB_6_, AB_8_, and AB_12_ samples. Detailed sample conditions and additional SEM images are provided in the Supporting Information (Table S2 and Figures S3–S10). Scale bars are 100 nm.

The initial mixing requires only 15 s of ultrasonication to form stable AB_*N*_-clusters. This condition enables interconnecting multidentate HB interaction to form polymer bundles between the central A-NP and each individual adjacent B-NP, akin to covalent bond formation in molecules. The resulting PM bonds also possess characteristics such as “bond length”, i.e., the interparticle distance between A- and B-NPs (*d*_A–B_), and “bond strength” correlating with the ratio of connected/unconnected HB pairs. When the freshly prepared PM colloid (Figure 1C) is drop-cast onto a substrate and left to dry, 2D PMs form (Figures 1D and 2A), being separated and with well-controlled coordination numbers *N*. The mechanism for such a 2D structure formation is illustrated in Figure 1C,D and thoroughly discussed in our recent study.^26^ Briefly, the capillary force between the central A-NP and the substrate surface pulls surrounding B-NPs toward the substrate. Concurrently, steric and electrostatic repulsive forces from the bulky polymer bundles ensure that high symmetry is maintained even after 2D rearrangement. For example, AB_4_ PM transforms from a tetrahedral geometry in the colloidal state into a square planar configuration upon drying.

**Figure 3:**
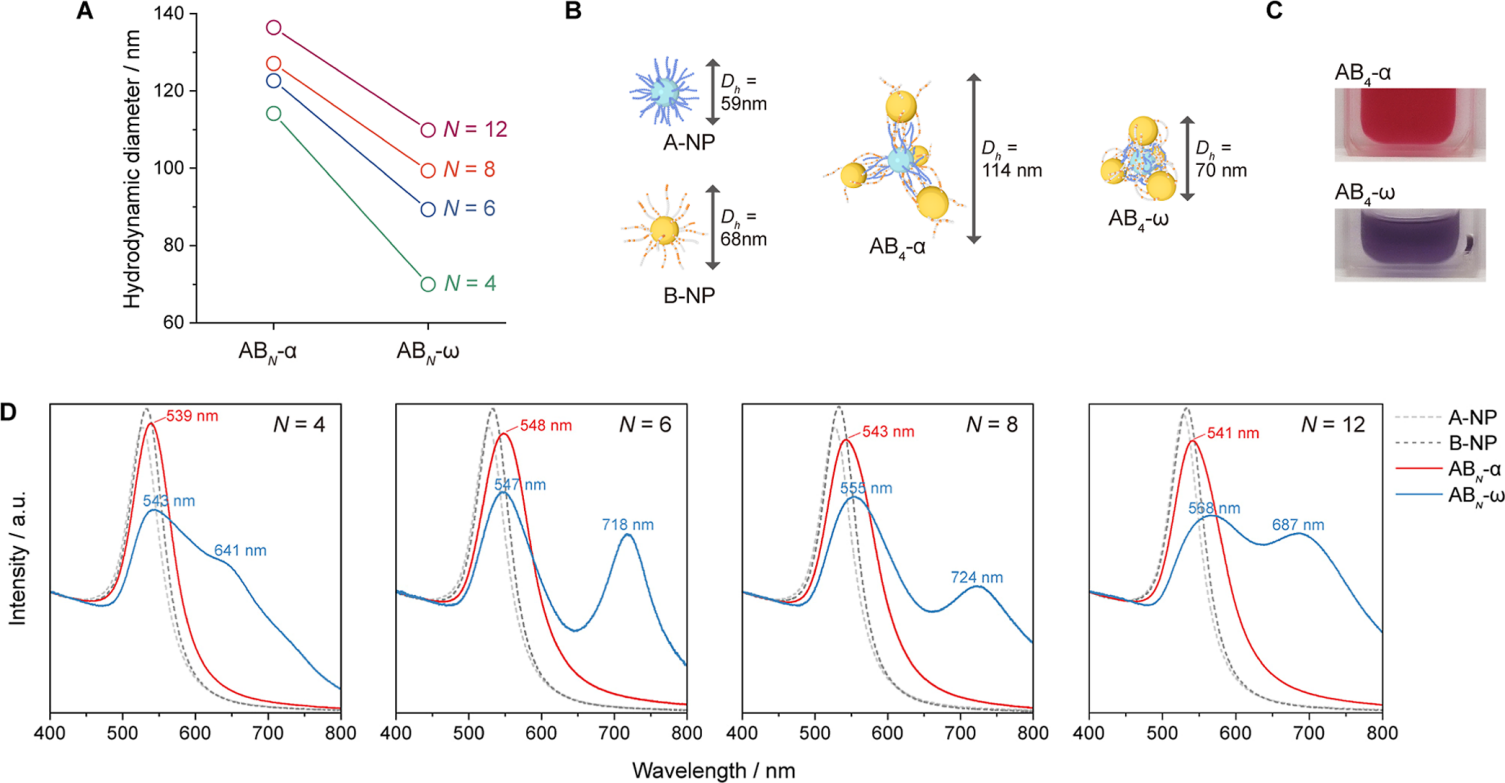
DLS and UV–vis results demonstrate the distinct colloidal behavior between AB_*N*_-α and AB_*N*_-ω and the significant effect of ultrasonication treatment. These colloids correspond to the samples used for preparing 2D/3D PMs’ isomers on substrates in Figure 2. (A) DLS measurements indicate that after post-ultrasonication, the hydrodynamic diameter (*D*_h_) of the PMs in the colloid is significantly reduced for all samples. (B) Schematic illustrations showing the changes in *D*_h_ from AB_4_-α to AB_4_-ω: after ultrasonication treatment, the *D*_h_ decreases from 114 to 70 nm, which is comparable with the size of pure B-NPs in the same solvent condition (68 nm). (C) Photograph of the AB_4_-α and AB_4_-ω colloids. The color shift from red to violet indicates strong plasmonic coupling due to the significantly decreased *d*_A–B_ within a cluster. (D) UV–vis spectra of AB_*N*_-α and AB_*N*_-ω for *N* = 4, 6, 8, and 12, compared with the spectra of A- and B-NPs under the same solvent conditions.

The PM’s colloidal structure should avoid the aforementioned 3D to 2D transition to create a stable tetrahedral isomer on the substrate. Therefore, the structural stiffness must be increased to prevent the collapse of the 3D geometry under capillary forces during the drying process. For this task, we aim to decrease the PM bond length and concurrently increase the bond strength to stabilize the PM structure. Experimentally, we found, to our surprise, that an additional ultrasonication step significantly changes the structure of the colloidal PMs: We will refer to the just-assembled colloid as the AB_*N*_-α state while to the ultrasound-treated as the AB_*N*_-ω state. In the AB_4_ sample, after post-ultrasonication for a few minutes (continuously) at room temperature and following incubation, the colloid’s color changes from red to violet (Figure 3C), indicating a drastically enhanced plasmonic coupling between the AuNPs^15^ within the colloidal PMs. DLS results further indicate a drastic reduction in the hydrodynamic diameter (*D*_h_) from 114 to 70 nm when going from the AB_4_-α to the -ω state (Figure 3B). This change is particularly impressive compared to the *D*_h_ of B-NPs, which is 68 nm and thus nearly identical to that of AB_4_-ω.

This dramatic state change in the colloidal PMs results from the altered interactions among HB-donor polymers, HB-acceptor polymers, and solvent molecules. Ultrasonication, acting as a high-frequency mechanical wave, clearly can impact the HB interaction,^42,43,50^ and Figure 1G shows a rational explanation for our case: In the AB_*N*_-α state, most HB moieties on the polymer are solvated with a few bonded to each other. The ultrasonication cleaves the bound solvent molecules from the corresponding HB moieties, temporarily causing desolvation and thus promoting new HB binding sites between polymers. Similar ultrasonication-induced desolvation phenomena have also been reported by Rahimzadeh et al. They observed that poly(*N*-isopropylacrylamide) exhibits an exceptionally high ultrasonication-responsiveness to dehydration at certain frequencies through the breakage of HB with water molecules. This effect occurs at a speed that even surpasses the well-known temperature-responsiveness of poly(*N*-isopropylacrylamide).^42,43^ In our case, a bath-type sonicator provides (by accident) the appropriate sonication conditions for our polymer/solvent system. Ultrasonication accelerates the PMs from the α state (more solvent–polymer HB) toward the ω state (more polymer–polymer HB). (Preliminary experiments with specialized ultrasound generators at different frequencies and power levels did not yield improved results; this will be the subject of future studies). Moreover, as the interchain linkages increase, the PMs become more rigid and the PM bond strength increases. Similar phenomena can be extensively found in studies where ultrasound stimuli are utilized for gelation by inducing cross-linking from HB and other noncovalent interactions.^51−54^ Consequently, the stabilized AB_4_-ω PM is able to maintain its tetrahedron structure when transferred from the colloid to substrate (Figure 1F).

Following solvent loss and a higher PM’s bond strength, the polymer bundles shrink, and the polymer chains entangle tightly, pulling the adjacent B-NPs toward the A-NP (Figure 1E). Consequently, the *D*_h_ decreases and stronger plasmonic coupling occurs.

Notably, the proportions of 2D or 3D isomer are 100% for each substrate sample, indicating that ultrasound facilitates a 100% yield of the α–ω transition and ensures the consistent evolution of each PM bond.

In short, the α–ω transition changes the properties of the PM from several perspectives, including the PM bond length, bond strength, and isomerization capability during drop-casting. Interestingly, these three properties are also decisive parameters for a real molecule. The relationship in which a short PM bond corresponds to a stronger bond strength parallels that seen in chemical bond systems. Although the mechanism of PM and molecular bond is fundamentally different, both systems share similarities for the formation of defined symmetry within an interacting AB_*N*_ ensemble.^55−57^

### 2D/3D Isomerization of PMs with Varied Coordination Numbers

With an understanding of the impact of ultrasound on the α–ω transition and the dynamic structural change of PMs on solid substrates, we aim to generalize the structural isomerization across a broad spectrum of coordination numbers. In addition to AB_4_, we choose *N* = 6, 8, and 12 due to their high symmetry in 3D configurations. PMs with target *N* number are successfully fabricated using our model of solvent-competing self-assembly,^26^ where we found that in a THF/CHCl_3_ mixture with *x*(THF) of 7.0–12.5%, the self-assembly of PMs proceeds in a controlled fashion. Within this concentration window, the P(St_0.7_-*r*-HSt_0.3_)_*m*_ polymer chains on B-NPs gradually collapse as the *x*(THF) decreases, causing more B-NPs to be able to pair with one A-NPs. As such, a single set of A-/B-NP can span several *N* values in assembling PMs by only slightly changing the solvent composition in 1–2% increments. Further variation can be introduced by altering the ratio of total phenol to ether groups, by changing either the polymer chain length or the particle size.^26^

For demonstration, we fixed the polymer length at *m* = 420 and the AuNP’s size at 34 nm on B-NPs and varied the polymer length or AuNPs’ sizes on A-NPs, with variations in the solvent ratio to obtain all targeted PMs. Specifically, PEG_136_-capped 22 nm A-NPs are used for AB_4_ (*x*(THF) = 10.0%); PEG_227_-capped 28 nm A-NPs are used for AB_6_ and AB_8_ (*x*(THF) = 10.0% and 7.5% respectively); PEG_454_-capped 30 nm A-NPs are used for AB_12_ (*x*(THF) = 7.0%). Detailed conditions are summarized in Table S2.

For all AB_*N*_ discussed herein, the AB_*N*_-α and AB_*N*_-ω states were produced using the above-mentioned method. These colloidal states were analyzed by using DLS (Figure 3A) and UV–vis spectroscopy (Figure 3D). Structural isomerization of these states on the substrate was examined by SEM (Figures 2 and S3–S10).

Figure 2C and Table S2 demonstrate that samples meet the target *N*-value. Figure 2A,B shows the successful formation of 2D/3D PMs’ isomers from their respective AB_*N*_-α and AB_*N*_-ω states without losing any adjacent B-NPs upon ultrasonication (statistical comparison for AB_4_ samples is shown in Figure S21 and Table S2). Analogue to the planar square/tetrahedron isomerization for AB_4_, AB_6_ features a hexagonal configuration for 2D and a nearly octahedral one for 3D. AB_8_’s 2D form appears as a deformed octagon as the circumference of A-NP is insufficient to accommodate all 8 B-NPs. Its 3D form predominantly exhibits a square antiprismatic geometry, where the upper four B-NPs are situated in the gaps of the lower layers, indicating a minor effect of capillary forces (with a few exceptions forming cubes). The AB_12_ samples exhibit the most extreme geometry change: The 2D form of AB_12_ visually shows a “splash” of B-NPs, including some B-NPs located on the second layer while remaining attached to the A-NPs. This supports the assumption that the PM bonds are extended in the colloidal AB_*N*_-α state. The structure exhibits high plasticity, allowing for the elongation of polymer bundles to the outermost B-NPs. As the PM bond length shortens and the overall structural stiffness increases, the AB_12_-ω state leads to a densely packed globular (3D) structure on the substrate. Notably, a perfect icosahedron with *hcp* stacking is formed when *N* is exactly 12.

DLS results (Figure 3A) confirm the significant reduction of *D*_h_ for all AB_*N*_ samples, indicating that all PMs undergo a similar degree of α–ω transition under ultrasonication. The UV–vis spectra provide clearer evidence of PM formation as well as changes in PM bond length (Figure 3D): The plasmonic resonance peak of the AB_*N*_-α colloid is close to that of single A-/B-NPs. A slight red shift here can be attributed to the increased surrounding refractive index due to self-assembly as the NPs come closer. The decreased *d*_A–B_ induced by ultrasonication leads to the AB_*N*_-ω state, which shows two characteristic resonance modes: one at 540–570 nm (λ_1_) and a new mode at 640–720 nm (λ_2_). For the low coordination numbers (*N* = 4, 6), λ_1_ can be associated with the resonance mode of a single AuNP.^15^ It shares the same position as that seen in the α state. The significant red shift of λ_2_ from the resonance mode of AuNPs can be attributed to the localized surface plasmon coupling between A- and B-NPs, where the A-NPs induce oppositely directed dipoles in the B-NPs.^15,58^ This hybridized mode suggests that the PM bonds are considerably shortened, as the degree of red shift in λ_2_ correlates with *d*_A–B_—the shorter the distance, the stronger the shift.^58^ We will further discuss this in the next section in greater detail. The influence of coupling between B-NPs in these cases is negligible due to the wide distance between B-NPs (detailed explanation, see Figure S19). For *N* = 8 and 12, as the distance between B-NPs significantly decreases, the B–B coupling begins to take effect, as evidenced via the simulation where the central A-NP is removed (Figure S20). This hybridization forms a bright superradiant collective mode and a dark subradiant collective mode (Fano resonance).^17,59^ Thus, the spectra of PMs are seen as a broad and continuous bright mode separated by a distinct dip from the dark mode due to destructive interference. As a result, this leads to a red-shift of the λ_1_ peak maximum and a broadening of the overall peaks, particularly evident in AB_12_ PMs.

### Spectroscopic Study of Ultrasonication Altered PMs’ Bond Lengths and Simulations

This section focuses on studying how ultrasonication affects the PM’s bond lengths and how these changes are reflected in spectroscopic results. We designed a series of experiments with a step-by-step (SBS) treatment to shorten the PM bond length gradually. This involved pausing between each ultrasonication session to record changes in the hydrodynamic diameter and absorption spectra. Simulations were also conducted to generate theoretical spectra with different *d*_A–B_. Comparing experimental and simulation results gives insights into the structural changes during the α–ω transition.

For these experiments, we selected the AB_6_ structure for demonstration. Both A-NPs and B-NPs were chosen with the same diameter (34 nm) to simplify the system and mitigate the effects caused by differences in the NP size. This series of samples is referred to as AB_6_^SBS^ to distinguish it from the previous AB_6_ samples.

**Figure 4:**
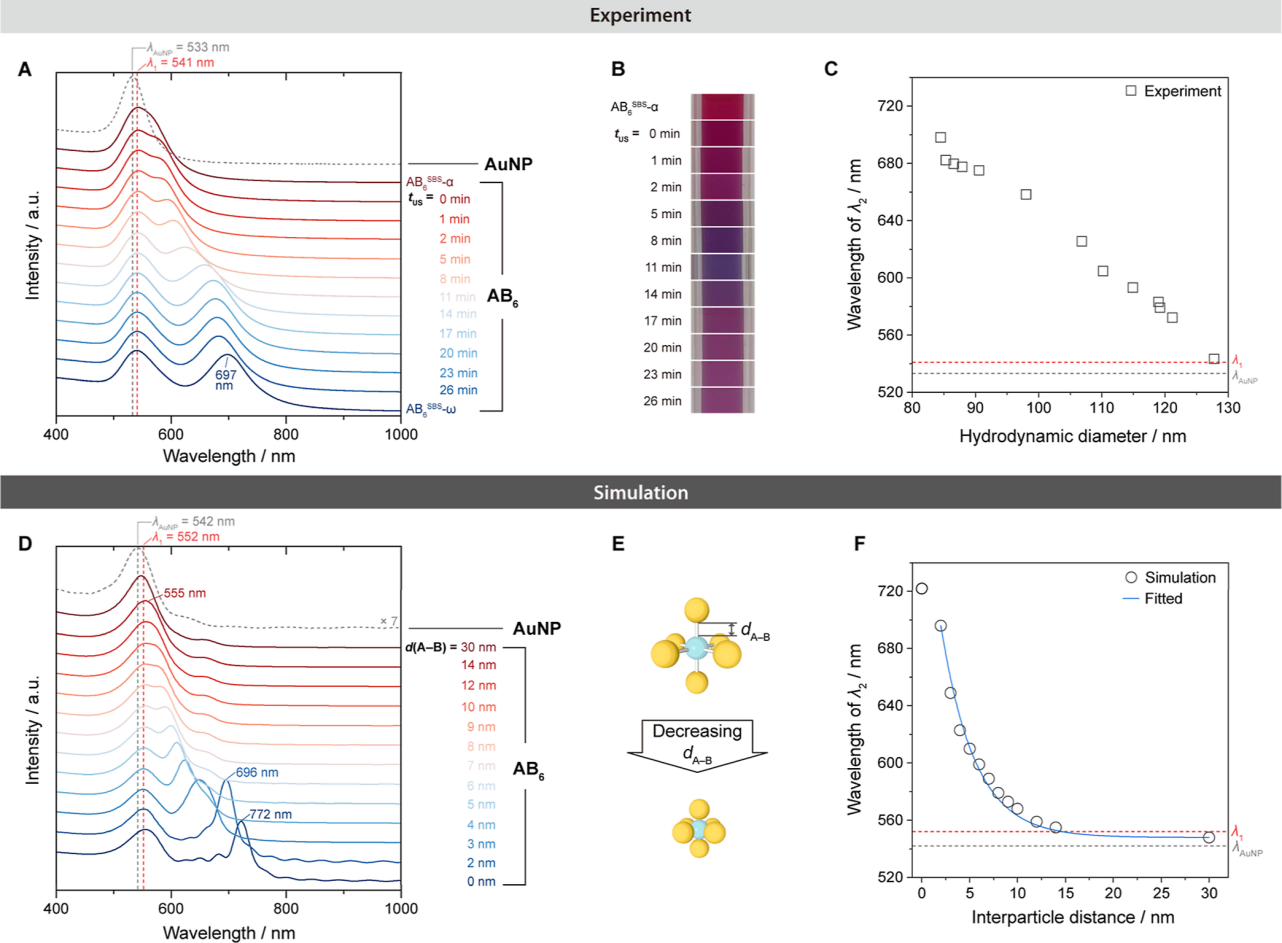
Experimental and simulation results of step-by-step ultrasonication on AB_6_^SBS^ colloid. (A–C) Experiment: (A) UV–vis spectra for B-NPs (gray dashed) and AB_6_^SBS^ colloid (red to blue) at each step. (B) Photographs showing color changes. (C) Relationship between λ_2_ peak position and hydrodynamic diameter (measured by DLS) with red-shift indicating PM shrinkage. Gray and red dashed lines show resonance modes of pure B-NPs and λ_1_, respectively. (D–F) Simulation: (D) simulated absorption spectra for AB_6_ structures with decreasing *d*_A–B_. The gray dashed curve is the spectrum of a single 34 nm AuNP multiplied by seven. (E) Schematic of *d*_A–B_ changes. (F) Relationship between λ_2_ wavelength and *d*_A–B_, with a fitted curve for *d*_A–B_ = 2–30 nm. Gray and red dashed lines indicate resonance modes of 34 nm AuNP and λ_1_, respectively.

As shown in Figure 4A–C, the fresh assembled sample (AB_6_^SBS^-α) was first recorded and then left to incubate overnight before recording again (AB_6_^SBS^(*t*_US_ = 0)) prior to ultrasonication steps. Each ultrasonication step lasted 1–3 min, with *t*_US_ representing the cumulative ultrasonication duration (AB_6_^SBS^(*t*_US_ = 1–26 min)). These steps were repeated, until only minor changes were observed in the UV–vis spectra. After 10 ultrasonication steps, the sample was incubated overnight again for the final recording (AB_6_^SBS^-ω).

Inspection of UV–vis spectra (Figure 4A) reveals the gradual evolution of two peaks: λ_1_, consistently located at 541 nm, predominantly originates from the resonance mode of AuNPs; λ_2_, which separates from λ_1_, exhibits red-shifting and increased intensity after each step. This behavior aligns well with the previous discussion, suggesting that λ_2_ is a hybrid mode enhanced by stronger plasmonic coupling due to the decreased *d*_A–B_. These spectral shifts correspond to the visual color changes in the sample, as depicted in Figure 4B: The colloid initially transitions from red to purple, attributed to the λ_2_ band moving to around 650 nm; then, it takes on a pink-red color as this hybrid mode further transitions toward a lower energy region (around 700 nm). The consistent shifting of λ_2_ while maintaining a narrow bandwidth, alongside apparent color changes, allows us to infer that the PM bond length changes are uniform across all of the PMs in the samples.

DLS measurements (Figure 4C) reveal a consistent decrease in *D*_h_ after each ultrasonication step, directly correlating with the shift of λ_2_. This affirms the fact that the PM’s shrinkage and spectroscopic evolution proceed simultaneously. This relationship persists across individual experiments with the same pattern (Figure S2B). We have repeated this sequential treatment of AB_6_^SBS^ PM with a 4-fold diluted particle concentration and observed the same trend but faster α–ω evolution under the same ultrasonication power condition (Figure S2A).

To further clarify the α–ω evolution, we incorporated two incubation phases (each about 16 h) before and after the experimental sequence of ultrasonication steps. Both UV–vis and DLS results indicate that the PMs also evolve slightly during these phases, albeit several magnitudes slower than when applying ultrasonication. This suggests that the ω state is thermodynamically more favored than the α state. Ultrasonication likely accelerates this process by overcoming the activation energy needed for solvent dissociation and the formation of new HB between polymers.

**Figure 5:**
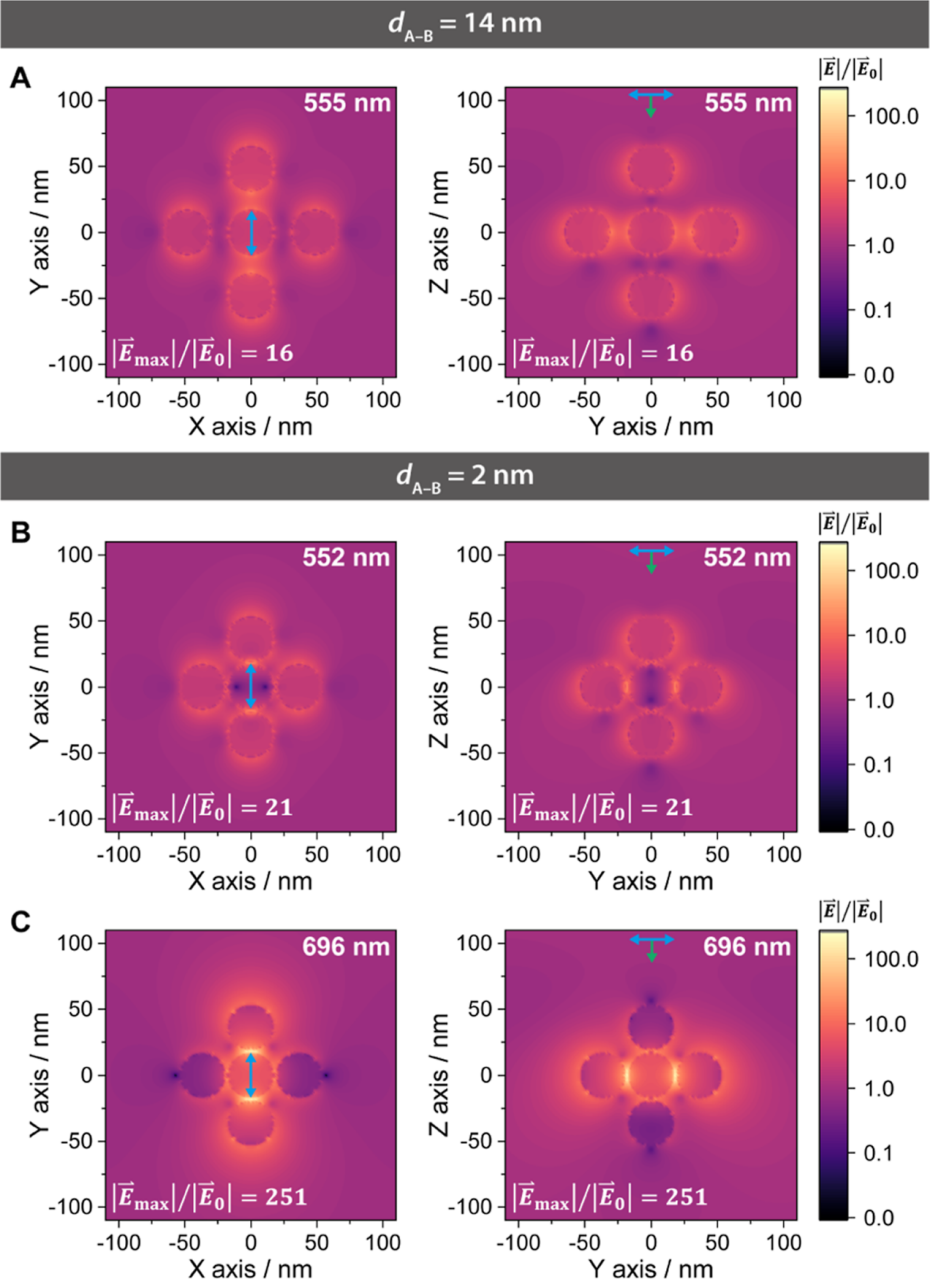
Electric field profiles at resonance modes for the *d*_A–B_ = 14 and 2 nm, displayed in both *xy* and *yz* planes for a colloidal AB_6_ PM (same model as in Figure 4). (A) Electric field profile for *d*_A–B_ = 14 nm at λ = 555 nm (spectral maximum), with a calculated enhancement factor of 16. (B,C) Electric field profiles for *d*_A–B_ = 2 nm at λ_1_ = 552 nm (B) and λ_2_ = 696 nm (C), with calculated enhancement factors of 21 and 251, respectively.

Furthermore, we have utilized our expertise in electromagnetic modeling via finite-difference time-domain (FDTD) simulation methods^60,61^ to determine the impact of changes in the PM’s bond length on spectral variations (Figure 4D–F) and field enhancement (Figure 5) under different PM bond length conditions. The simulation model (Figure S13) closely replicates the experimental conditions of the AB_6_^SBS^ series, that is, an AB_6_ structure with 34 nm A-NPs and B-NPs covered with the corresponding polymer shell, configured in octahedral coordination and aligned with the XYZ axes of the coordinate system. The *d*_A-B_ is defined as the edge-to-edge distance between the gold surface of A- and B-NPs (Figure 4E) and varies from 30 to 0 nm.

The simulated absorption cross-section spectra (Figure 4D) exhibit consistency with the experimental spectra: (1) λ_1_ slightly red-shifts from the single AuNPs and stabilizes at 552 nm across all *d*_A–B_ values. Minor deviations from the experimental data (541 nm) are likely due to approximations in the polymer shells modeling. (2) λ_2_ is near absent at *d*_A–B_ = 30 nm and then begins to appear as a peak broadening at *d*_A–B_ = 14 nm. It continues red-shifting and increases in intensity, moving to 696 nm at *d*_A–B_ = 2 nm (close to the position of the AB_6_^SBS^-ω sample). If *d*_A–B_ is shortened to 0 nm, λ_2_ reaches its theoretically highest shift to 722 nm. More quantitatively, the wavelength of λ_2_ shows a near-exponential decay with distance (Figure 4F), in line with the universal scaling behavior of surface plasmon coupling in dimers,^58,62^ and can be described with an empirical surface plasmon ruler equation
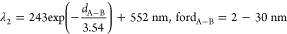


The electric field profiles (Figure 5) further confirm the origins of the λ_1_ and λ_2_ modes. For λ_1_, no significant coupling or changes in the field enhancement factor are observed, even as *d*_A–B_ decreases to 2 nm, indicating its derivation predominantly from AuNPs (Figure 5A,B). In contrast, λ_2_ = 696 nm exhibits significant “hot spots” between A-NPs and B-NPs, with an enhancement factor reaching 251 at *d*_A–B_ = 2 nm (Figure 5C). This illustrates its dependence on strong plasmonic coupling between A- and B-NPs.

From the experimental and simulation findings, we have pinpointed that the spectra for *d*_A–B_ at 14 and 2 nm align most closely with the AB_6_^SBS^-α and AB_6_^SBS^-ω states, respectively (Figure 4A,D). Despite some deviations, we can still infer that transitioning from the α state to the ω state requires a reduction of >10 nm in *d*_A–B_ and that ultrasonication facilitates a significant shrinkage down to a few-nm interparticle distances. This fact strongly supports our interpretation and gives a comprehensive visualization of the significant change in PM bond properties during α–ω evolution. The predicted plasmonic hot spots also reveal a strong potential to apply ω-PM for sensing and optical applications.

## Conclusions

In summary, we developed an HB-based platform for the self-assembly of polymer-grafted AuNPs into a series of colloidal PMs. The coordination number of these PMs can be tailored by adjusting the polymeric building blocks, the nanoparticle dimensions, and the self-assembly conditions. Ultrasonication is found to be an exceptionally effective and controllable stimulus that transforms colloidal PMs from an α-state with long and flexible bonds to an ω-state with short bonds and a rigid structure, as evidenced by SEM, UV–vis spectra, and DLS results. The colloidal state of the PM decides its configuration on a solid substrate after casting: α-state PMs are arranged in 2D isomers, while ω-state PMs form 3D isomers. Unlike the current methods in the literature that require complex redesigns of assembly pathways, our method overcomes this by using a simple ultrasonication process. Additionally, step-by-step ultrasonication experiments and FDTD simulations detail the progressive α–ω transition by bridging spectroscopic behaviors with the gradual change in the bond length, *d*_A–B_. From the calculated plasmon ruler from the simulations, the *d*_A–B_ for α- and ω-states can be well estimated as 14 and 2 nm. Our approach offers a rational design and the tailored construction of nanoarchitectures. The tunable optical behavior of the PMs that may be dialed up by easily controlling their bond lengths provides great potential for the fabrication of optical devices and sensors.

## Experimental Section

### Synthesis of Thiol-Terminated P(St_0.7_-*r*-HSt_0.3_)_*m*_

The synthesis of thiol-terminated P(St_0.7_-*r*-HSt_0.3_)_*m*_ followed previously reported procedures.^26^ As shown in the synthetic route (Figure S1A), this two-step process begins with the synthesis of a benzodithioate-terminated random copolymer of styrene and 4-acetoxystyrene, P(St_0.7_-*r*-ASt_0.3_)_*m*_, through RAFT polymerization. Subsequently, the polymer was hydrolyzed to form thiol-terminated P(St_0.7_-*r*-HSt_0.3_)_*m*_.

In brief, benzyl benzodithioate (3.4 mg, 0.014 mmol), styrene (1.46 g, 14.0 mmol), and 4-acetoxystyrene (973 mg, 6.00 mmol) were combined in a glass vial. The mixture was purged with argon for 15 min to ensure an inert environment. The reaction was performed at 110 °C for 18 h and then quenched by exposing the mixture to air and rapidly cooling it in an ice bath. The polymer was purified through four cycles, each consisting of precipitation with methanol, collection by centrifugation, and redispersion in THF. The obtained benzodithioate-terminated P(St_0.7_-*r*-ASt_0.3_)_*m*_ was dried in a vacuum at 100 °C overnight. For hydrolysis, 1 g of the as-obtained intermediate polymer was dissolved in 20 mL of dioxane in a glass vial, which was then purged with argon. After hydrazine (2 mL) was injected, the mixture was shaken overnight. The polymer underwent three cycles of purification, each involving precipitation in hexane, centrifugation, and redispersion in THF. Finally, the thiol-terminated P(St_0.7_-*r*-HSt_0.3_)_*m*_ was dried in a vacuum oven at 130 °C overnight.

From the SEC measurement (Figure S1B), the average molecular weight *M̅*_*n*_ of benzodithioate-terminated P(St_0.7_-*r*-ASt_0.3_)_*m*_ was determined to be 50.7 kg/mol with a dispersity *D̵* = 1.2. ^1^H NMR was used to determine the polymer composition (ratio between St/ASt, and St/HSt) and confirmed that the hydrolysis process is complete (Figure S1C). From these characterizations, the degree of polymerization was calculated to be *m* = 420.

### Synthesis of AuNPs

CTAC-stabilized spherical AuNPs were prepared using the method reported by Xia’s group,^63^ with an up-scaling of the feed ratio. This method involves first preparing cetyltrimethylammonium bromide (CTAB)-stabilized single crystal Au-clusters followed by two subsequent growth steps: the first to ∼10 nm to form CTAC-stabilized seeds and the second to achieve the final size of the AuNPs. Briefly, the cluster was prepared by rapidly adding a NaBH_4_ aqueous solution (0.6 mL, 10 mM, freshly dissolved) to a mixture of HAuCl_4_ (5 mL, 0.5 mM) and CTAB (5 mL, 200 mM) under vigorous stirring. The mixture was stirred for another 15 min at 27 °C and left undisturbed at 27 °C for 3 h. The ∼10 nm seed is prepared by rapidly adding a solution of HAuCl_4_ (50 mL, 0.5 mM) into the mixture of CTAC (50 mL, 200 mM), ascorbic acid (37.5 mL, 100 mM), and 1.25 mL cluster at 27 °C. After stirring for another 15 min, the seeds were collected and washed once with water through ultracentrifugation at 10^5^*g* and redispersed in 22.5 mL of 20 mM CTAC for the next step. For the second growth step, CTAC (1040 mL, 100 mM), ascorbic acid (67.6 mL, 10 mM), and the as-prepared ∼10 nm seeds were mixed in a container. The final size of the AuNPs is controlled by the amount of the seeds. A syringe pump system was used to continuously add a HAuCl_4_ aqueous solution (1040 mL in total, 0.5 mM) into the mixture with an injection rate of 1040 mL/h while stirring. After the injection was completed, the mixture was stirred for an additional 15 min to ensure the completion of the reaction. The AuNP product was collected and washed twice with 1 mM CTAC and finally redispersed in 1 mM CTAC as a stock dispersion with an approximate concentration of 8 mg[Au^0^]/mL.

### Preparing A-NPs and B-NPs by Polymer Functionalization on AuNPs

The polymer functionalization on AuNPs followed the procedure previously reported,^26^ with some modifications. Typically, 1 mL of a stock dispersion of CTAC-stabilized 34.3 nm AuNPs was rapidly added to a polymer solution of P(St_0.7_-*r*-HSt_0.3_)_420_ in THF under sonication. The sonication continued for 30 min, after which the mixture was left undisturbed overnight before purification. The excess polymer was removed through 13 cycles of centrifugation/redispersion in THF. Notably, these multiple washing cycles are crucial for removing any trace amounts of unbound polymer, which can disturb the self-assembly result. Finally, the P(St_0.7_-*r*-HSt_0.3_)_420_-functionalized AuNPs were redispersed in 1 mL of THF as a stock dispersion, with an approximate concentration of 8 mg[Au^0^]/mL. All A-NPs and B-NP used in this work, along with the specific conditions for each sample, are summarized in Table S1.

### Assembling A- and B-NPs into AB_*N*_-α State of PMs

For assembling AB_*N*_-α (*N* = 4, 6, 8, 12) PMs, the stock colloid of B-NPs (10 μL, in THF) was diluted to 300 μL (B-sol) in a 2 mL glass vial. The stock colloid of A-NPs (predetermined amount, in THF) was diluted into 100 μL (A-sol) in a polypropylene vial. Both A-sol and B-sol were adjusted during the dilution with the necessary volumes of THF and CHCl_3_ to achieve the required solvent compositions. For the sample that was used later for the AB_6_^SBS^ experiment series, a 4-fold scale was used. The initial mixing process involved adding A-sol to the B-sol under bath sonication, with the mixture sonicated for 15 s (Movie S1). For the AB_*N*_-α state, DLS measurements, UV–vis spectroscopy, and sample preparation for SEM measurements were conducted with the freshly prepared PMs colloid. The sample colloid can be stored in a perfluoroalkoxy alkane vial to avoid the loss of PMs through adsorption onto the glass surface. The detailed information on all PMs used in this work is summarized in Table S2.

Post-ultrasonication for transitioning AB_*N*_-α to AB_*N*_-ω PMs for 3D structures on the substrate. To convert AB_*N*_-α PMs (where *N* = 4, 6, 8, 12) into AB_*N*_-ω PMs for 3D structures formation on the substrate, 100 μL of the overnight-incubated PMs colloid was placed in a 2 mL glass vial. This vial was then positioned at the center of an ultrasonication bath (Figure S22 and Movie S2). The ultrasonication process was continued until the hydrodynamic diameter of the PMs was reduced by about 20–25 nm. It is important to note that ultrasonication efficiency is highly dependent on diverse factors, including the colloid volume and concentration. For the given volume and concentration, sonication duration of 5, 5, 2, and 3.5 min was found to be the optimal condition for AB_4_, AB_6_, AB_8_, and AB_12_ samples, respectively. Then, the colloid was transferred into a PFA vial and left undisturbed overnight to allow for the complete formation of AB_*N*_-ω PMs. DLS measurements and UV–vis spectroscopy were performed on AB_*N*_-ω PMs to obtain their hydrodynamic diameter and absorption spectra in colloids. For visualizing their 3D geometry on the substrate, sample colloids were drop-cast onto a silicon substrate and used for SEM measurements.

Step-by-step ultrasonication experiments were performed with AB_6_ structures (AB_6_^SBS^ series). The AB_6_^SBS^ sample was used for the step-by-step ultrasonication experiment, which consisted of A-NPs and B-NPs with the same diameter of 34.3 nm. After the initial mixing of the A-NPs and B-NPs (AB_6_^SBS^-α), the colloid was incubated overnight prior to ultrasonication experiments. For the ultrasonication experiments, 700 μL of the overnight-incubated colloid was placed in a 7 mL glass vial (⌀ 16 mm) positioned at the center of the sonication bath. The sonication process began with a 1 min session for the first step, followed by 3 min for each subsequent step, accumulating a total sonication time of 26 min. After each sonication session, the colloid was left undisturbed for about 30 min to allow for the shrinking of the structure. DLS measurements, UV–vis spectroscopy, and photographs were taken before proceeding to the next ultrasonication step. This series of ultrasonication was continued until the changes observed in the hydrodynamic diameter and the absorption spectra were minor. The sample was incubated overnight to form AB_6_^SBS^-ω before the last measurements. In a separate individual experiment series, 700 μL of a 4-fold diluted colloid was used. This series involved 10 ultrasonication steps, totaling 11 min of accumulated sonication time, to achieve the same level of sonication efficiency as described above. The DLS results and UV–vis spectra from this series are shown in Figure S2.

### FDTD Analysis

FDTD-based simulations are carried out using a commercial-grade electromagnetic solver^64^ (Ansys Lumerical FDTD: 3D Electromagnetic Simulator, version 8.16). To reproduce the Mie scattering-based optical responses of the 3D PMs (AB_*N*_; *N* = 6) immersed in organic solvent (9% THF/91% CHCl_3_), “total-field scattered-field” (TFSF) sources are implemented with PML boundary conditions along the *X*, *Y*, and *Z* coordinates with an FDTD background refractive index of 1.4429. According to the experimental conditions of the AB_6_^SBS^ series, the diameters of the spherical AuNPs are considered to be 34.3 nm, with an additional polymer shell of 2 nm for both A-NP and B-NPs. The corresponding modeling is shown in Figure S13. The dielectric characteristics of gold are modeled using a six-coefficient data fit from Johnson and Christy,^65^ with an RMS error of 0.2. Additionally, the refractive index of the PEG shell for the A-NP is assumed to be constant, with values of 1.467. The refractive index of P(St_0.7_-*r*-HSt_0.3_)_420_ was approximated at 1.59 using the values for polystyrene. The absorption cross sections are calculated using the “analysis group”, placed inside the TFSF source, by considering the net power flow into the PM. To investigate the influence of the interparticle distance on the optical characteristics, the edge-to-edge distance between the gold surfaces of A- and B-NP (*d*_A–B_) is varied in discrete steps. Furthermore, to ensure the effect of plasmonic coupling, electric field profiles for the resonant modes are achieved by placing “frequency domain field and power” monitors across the diametric cross-section of the PM. An additional mesh grid with dimensions d*x* = 1 nm, d*y* = 1 nm, and d*z* = 1 nm is applied throughout all of the calculations for enhanced precision. Additional simulations for AB_6_, AB_8,_ and AB_12_ are also carried out to investigate the effect of B–B coupling through the removal of central A-NP (Figures S19 and S20).

## Supporting Information Available

Details of chemicals, characterization methods, and equipment; detailed polymer functionalization conditions for each A-NP and B-NP sample; detailed self-assembly conditions for each PM sample; coordination number statistics; synthesis route, SEC results, and ^1^H NMR spectra of the synthesized polymer; UV–vis and DLS results from an additional AB_6_^SBS^ experimental series; additional SEM images; modeling used for FDTD-based simulations; TEM images of A-NPs and B-NPs; additional simulation results; ultrasonication setup; and thermogravimetric analysis results (PDF)
Initial ultrasonication process to assemble A- and B-NPs into PMs (AVI)
Post-ultrasonication process (AVI)

## Supplementary Material

nn4c10912_si_001.pdf

nn4c10912_si_002.avi

nn4c10912_si_003.avi

## Abbreviations

NP(s): nanoparticle(s)
AuNP(s): gold nanoparticle(s)
PM(s): plasmonic molecule(s)
HB: hydrogen bond(ing)
RAFT: reversible addition–fragmentation chain-transfer
P(St_0.7_-*r*-HSt_0.3_)_*m*_: random copolymer of styrene and hydroxystyrene
PEG_*n*_: poly(ethylene glycol) methyl ether
CTAC: cetyltrimethylammonium chloride
CTAB: cetyltrimethylammonium bromide
DLS: dynamic light scattering
SEM: scanning electron microscopy
THF: tetrahydrofuran
*D*_h_: hydrodynamic diameter
AB_*N*_-α: just-assembled PMs in colloid
AB_*N*_-ω: ultrasound-treated PMs in colloid
*N*: coordination number
*d*_A–B_: the interparticle distance between A-NP and B-NP in a PM
SBS: step-by-step
FDTD: finite-difference time-domain
P(St_0.7_-*r*-ASt_0.3_)_*m*_: a random copolymer of styrene and 4-acetoxystyrene
