# Supplementary material for: Ultrasonic Control of Polymer-Capped Plasmonic Molecules
Source: ACS Nano. 2024 Oct 31;18(45):31360–71. doi: 10.1021/acsnano.4c10912 (PMC11562790; doi:10.1021/acsnano.4c10912)
Supplement: Supplementary file 1 — nn4c10912_si_001.pdf [file nn4c10912_si_001.pdf]

Supporting Information

Ultrasonic Control of Polymer-capped Plasmonic Molecules

Yingying Cai[a],*, Swagato Sarkar[b], Yuwen Peng[a], Tobias A.F. König[b,c,d], Philipp Vana[a]

[a] Institut für Physikalische Chemie, Georg-August-Universität Göttingen, Tammannstrasse 6, 37077 Göttingen, Germany.

[b] Leibniz-Institut für Polymerforschung Dresden e. V., Hohe Straße 6, 01069 Dresden, Germany

[c] Center for Advancing Electronics Dresden (cfaed), Technische Universität Dresden, Helmholtzstraße 18, 01069 Dresden, Germany

[d] Faculty of Chemistry and Food Chemistry, Technische Universität Dresden, Bergstraße 66, 01069 Dresden, Germany

*Corresponding Author: yingying.cai@uni-goettingen.de

Table of contents

1. Chemicals 1

2. Characterizations and equipment 1

3. Supplementary tables 3

4. Supplementary figures 5

5. References 27

1. Chemicals

Styrene, 4-acetoxystyrene, benzyl benzodithioate, hydrazine, cetyltrimethylammonium bromide (CTAB), cetyltrimethylammonium chloride (CTAC), $NaBH_4$, and ascorbic acid were purchased from Sigma-Aldrich. $HAuCl_4 \cdot 3H_2O$ (99.9%) was purchased from abcr GmbH. Tetrahydrofuran (THF), dimethylformamide (DMF), chloroform, methanol, dioxane, and hexane were purchased from VWR. Poly(ethylene glycol) methyl ether (PEG) thiol with $\overline{M}_{\mathrm{n}}$ = 6,000 g/mol was purchased from Sigma-Aldrich. Poly(ethylene glycol) methyl ether thiol with $\overline{M}_{\mathrm{n}}$ = 10,000 g/mol and $\overline{M}_{\mathrm{n}}$ = 20,000 g/mol was purchased from Biochem PEG. Prior to their use, styrene and 4-acetoxystyrene were purified with inhibitor remover. Ultrapure (type I) water (conductivity 18.2 MΩ · cm) was used for all experiments. All other chemicals were used as received.

2. Characterizations and equipment

Transmission electron microscopy (TEM). TEM measurements were conducted with a Philips CM 12 electron microscope equipped with an Olympus 1376 × 1032 pixel CCD camera. The sample was prepared by dropping 5 μL of colloid onto the TEM grid and left to dry in ambient conditions.

Scanning electron microscopy (SEM). SEM was carried out on an FEI Nova Nano SEM 650 microscope with an acceleration voltage of 15 kV and a working distance of 5 mm. For the sample preparation, 0.5 μL of freshly prepared colloid of assembled PMs was dropped onto a silicon wafer and dried with gentle argon flow.

Dynamic light scattering (DLS). DLS measurements were conducted using a Malvern Zetasizer Nano S system operating with a 633 nm laser at a scattering angle of 173° and a temperature of 25 °C. To perform these measurements, 10 μL of the PMs colloid was diluted with 1 mL of the corresponding THF/$CHCl_3$ mixture in a 1 cm thick glass cuvette. For A-NPs/B-NPs, 1.25 μL of their stock THF dispersion was diluted with the THF/$CHCl_3$ mixture to achieve the same solvent composition as used for the corresponding PMs. To determine the intensity-weighted mean hydrodynamic size (Z-average, i.e., D_h), it was necessary to know the viscosity constant and the refractive index of the corresponding THF/$CHCl_3$ solvent mixture.[1] The viscosity was calculated using a fitting equation from the literature.[2] The refractive index was measured with a Krüss DR6000-T refractometer, as cited in our previous work.[3] Each sample was measured three times to obtain an average value.

UV-Vis absorption spectroscopy. UV-Vis absorption spectroscopy was performed using a Jasco V770 scanning spectrophotometers. The sample colloid was analyzed in a 2 mm thick quartz cuvette, with baseline subtraction performed using the identical solvent mixture. The sample colloid can be diluted further with the identical solvent mixture if necessary. All spectra in this work are normalized at a wavelength of 400 nm for the same $Au^{[0]}$ concentration.[4]

Size-exclusion chromatography (SEC). SEC measurements were performed to determine the molecular weight of benzodithioate-terminated $P(St_{0.7}\text{-}r\text{-}ASt_{0.3})_m$ from RAFT polymerization. The SEC measurements were carried out at 35 °C using an Agilent 1260 Infinity SEC system equipped with a PSS GRAM precolumn (8 × 50 mm; styrene-divinylbenzene copolymer network; 5 μm particle size), three PSS GRAM separation columns PSS SDV; 8 × 300 mm; 10^6, 10^5, and 10^3 Å pore sizes), and an Agilent refractive index detector. The polymer was dissolved in THF with a 5 mg/mL concentration and an additional 0.1% toluene as the internal standard. THF was used as the eluent, with a 1.0 mL/min flow

rate. The SEC system was calibrated using low-dispersity linear polystyrene standards. The obtained molecular weight distribution is shown in **Figure S1B**.

^{1}H NMR. ^{1}H NMR spectroscopy was utilized to determine the molar ratios of acetoxy groups in P(0.7-*r*-$ASt_{0.3})_m$ and hydroxy groups in $P(St_{0.7}$-*r*-$HSt_{0.3})_m$. The polymer samples were dissolved in acetone-d^6 and measured using a Brucker Avance III 300 operating at a frequency of 300 MHz. The obtained spectra and the corresponding evaluation are presented in **Figure S1C**.

Ultrasonication. A bath-type ELMA S30H ultrasonic cleaner operating at 37 kHz with an effective sonication power of 80 W and peak performance maximum of 32 W was used. The ultrasonication treatment was conducted at room temperature, and the bath water was replaced between each session to prevent temperature increases. No significant temperature rise was observed, as the sonication time for each session did not exceed 5 minutes.

Thermogravimetric analysis (TGA). The TGA measurements were conducted using a TG 209 F3 Tarsus analyzer. Approximately 0.5 mL of the stock colloid of A-NPs or B-NPs was mixed with hexane to precipitate the polymer-capped AuNPs. The NPs were collected by centrifugation and dried in a vacuum oven to completely remove the solvent. The dried sample (ca. 2–4 mg) was transferred to a ceramic crucible for the TGA measurement, which was heated from 25°C to 1000°C at a heating rate of 10°C/min under a nitrogen gas flow of 20 mL/min. A baseline correction was conducted by heating an empty crucible using the same measurement procedure. The results are shown in **Figure S23**. The capping density (σ, in units of chains/nm^2) of the polymer on the AuNPs was estimated using the following **Equation S1**:

$$\sigma = \frac{f}{1-f} \cdot \frac{N_A \cdot \rho_{\text{Au}} \cdot d_{\text{AuNPs}}}{6\overline{M}_{\text{n}}},$$

where f is the total weight loss fraction, N_A is Avogadro constant, ρ_{Au} is the density of bulk gold, d_{AuNPs} is the average diameter of the AuNPs, and $\overline{M}_{\text{n}}$ is the average molecular weight determined by SEC.

3. Supplementary tables

Table S1. A-NPs and B-NPs used in this work, their polymer functionalization conditions, and purification conditions.

Polymer-functionalized AuNPs		Functionalization conditions		Purification conditions	Corresponding TEM images
		AuNP sol	Polymer solution		
A-NPs	PEG$_{136}$-grafted 22 nm AuNPs	1 mL of 21.7 ± 1.4 nm AuNPs in 1 mM CTAC	20 mL of 0.05 mM thiol-terminated PEG$_{136}$ in DMF	Washed 6 times with DMF, then with THF for remaining wash cycles	Figure S14
	PEG$_{227}$-grafted 28 nm AuNPs	1 mL of 27.5 ± 0.9 nm AuNPs in 1 mM CTAC	20 mL of 0.25 mM thiol-terminated PEG$_{227}$ in DMF	Washed 6 times with DMF, then with THF for remaining wash cycles	Figure S15
	PEG$_{454}$-grafted 30 nm AuNPs	1 mL of 30.3 ± 2.1 nm AuNPs in 1 mM CTAC	20 mL of 0.25 mM thiol-terminated PEG$_{454}$ in DMF	Washed 6 times with DMF, then with THF for remaining wash cycles	Figure S16
	PEG$_{227}$-grafted 34 nm AuNPs	0.25 mL (4 times concentrated) 34.3 ± 2.1 nm AuNPs in 0.1 mM CTAC	20 mL of 0.25 mM thiol-terminated PEG$_{227}$ in DMF	Washed 6 times with DMF, then with THF for remaining wash cycles	Figure S17
B-NPs	P(St$_{0.7}$-*r*-HSt$_{0.3}$)$_{420}$-grafted 34 nm AuNPs	0.25 mL (4 times concentrated) 34.3 ± 2.1 nm AuNPs in 0.1 mM CTAC	20 mL of 1 mg/mL thiol-terminated P(St$_{0.7}$-*r*-HSt$_{0.3}$)$_{420}$ in THF	Washed 13 times with THF	Figure S18

Table S2. Summary of PMs made in this work, including fabrication conditions, average coordination number, and SEM images.

**Sample name**	**A-NPs & B-NPs**	**x(THF) in THF/$CHCl_3$ mixture**	**Average coordination number**	**SEM images of 2D PMs**	**SEM images of 3D PMs**
AB_4-α	PEG_{136}-grafted 22 nm A-NPs<br>P($St_{0.7}$-*r*-$HSt_{0.3}$)$_{420}$-grafted 34 nm B-NPs	10.0%	3.9 ± 0.5 (determined from SEM images of 2D state)	Figure 1D<br>Figure 2A (column 1)<br>Figure S3	-
AB_4-ω			3.9 ± 0.5 (determined from SEM images of 3D states)	-	Figure 1F<br>Figure 2B (column 1)<br>Figure S4
AB_6-α	PEG_{227}-grafted 28 nm A-NPs<br>P($St_{0.7}$-*r*-$HSt_{0.3}$)$_{420}$-grafted 34 nm B-NPs	10.0%	5.8 ± 0.7	Figure 2A (column 2)<br>Figure S5	-
AB_6-ω				-	Figure 2B (column 2)<br>Figure S6
AB_8-α	PEG_{227}-grafted 28 nm A-NPs<br>P($St_{0.7}$-*r*-$HSt_{0.3}$)$_{420}$-grafted 34 nm B-NPs	7.5%	7.8 ± 0.8	Figure 2A (column 3)<br>Figure S7	-
AB_8-ω				-	Figure 2B (column 3)<br>Figure S8
AB_{12}-α	PEG_{454}-grafted 30 nm A-NPs<br>P($St_{0.7}$-*r*-$HSt_{0.3}$)$_{420}$-grafted 34 nm B-NPs	7.0%	12.2 ± 1.4	Figure 2A (column 4)<br>Figure S9	-
AB_{12}-ω				-	Figure 2B (column 4)<br>Figure S10
AB_6^{SBS} series	PEG_{227}-grafted 34 nm A-NPs<br>P($St_{0.7}$-*r*-$HSt_{0.3}$)$_{420}$-grafted 34 nm B-NPs	9.0%	5.6 ± 0.7	AB_6^{SBS}-α:<br>Figure S11	AB_6^{SBS}(t_{US} = 20 min):<br>Figure S12

4. Supplementary figures

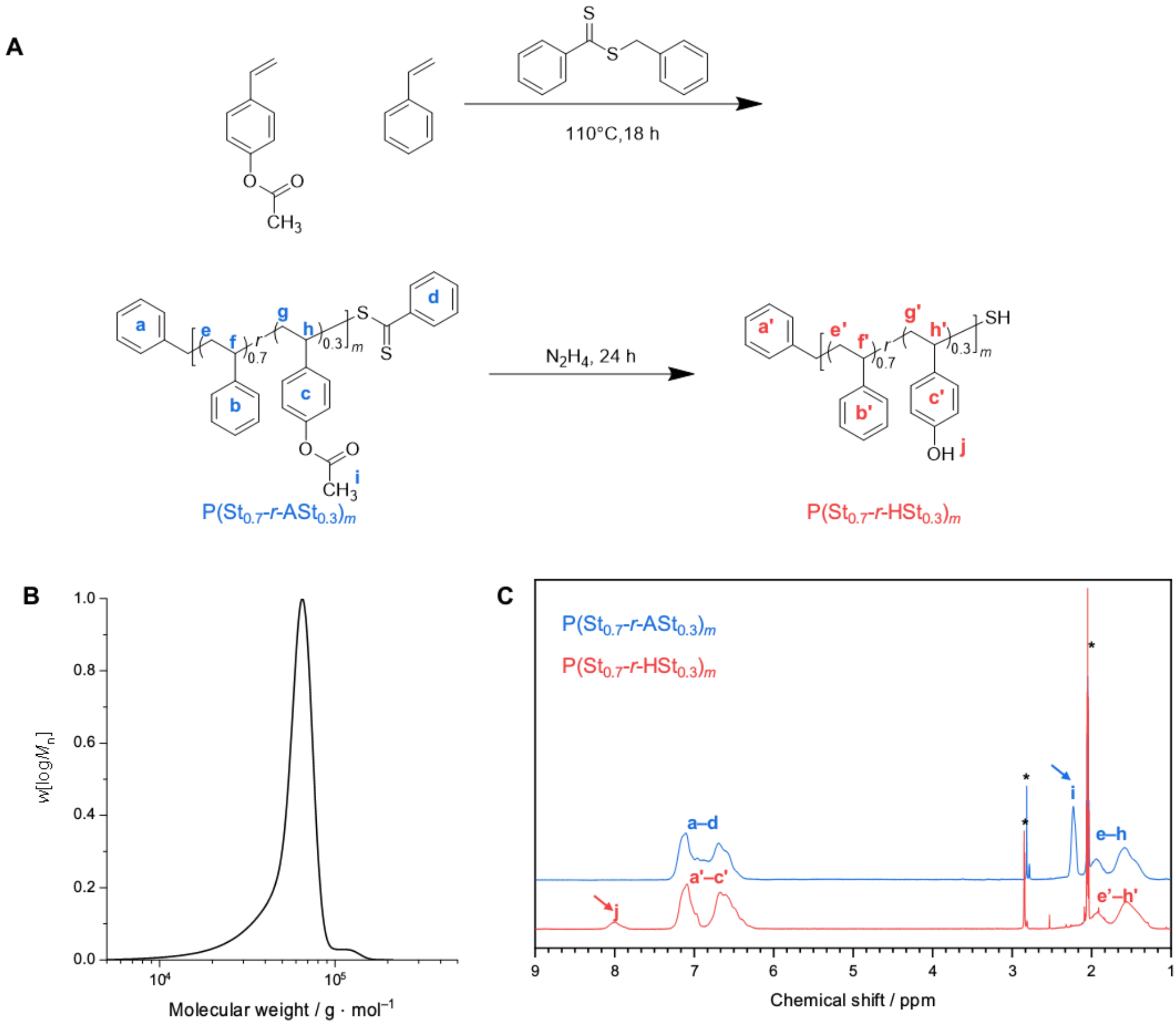

Figure S1. (A) Synthetic route of benzodithioate-terminated P(St$_{0.7}$-*r*-ASt$_{0.3}$)$_m$ *via* RAFT polymerization followed by a one-step hydrolysis process to form thiol-terminated P(St$_{0.7}$-*r*-HSt$_{0.3}$)$_m$. Alphabetic markers indicate the corresponding peaks in the ^{1}H NMR spectra. **(B)** Molecular weight distribution of benzodithioate-terminated P(St$_{0.7}$-*r*-ASt$_{0.3}$)$_m$ obtained by SEC measurement. **(C)** ^{1}H NMR spectra of P(St$_{0.7}$-*r*-ASt$_{0.3}$)$_m$ and P(St$_{0.7}$-*r*-HSt$_{0.3}$)$_m$ measured using acetone-d^6 as the solvent. Asterisks indicate the solvents' peak. The polymer composition (molar ratio between St/ASt, and between St/HSt) was calculated with the ratio between the integrals of peak i and peaks a–d, as well as between peak j and peaks a'–c'. The peaks i and j indicate a complete hydrolysis result.

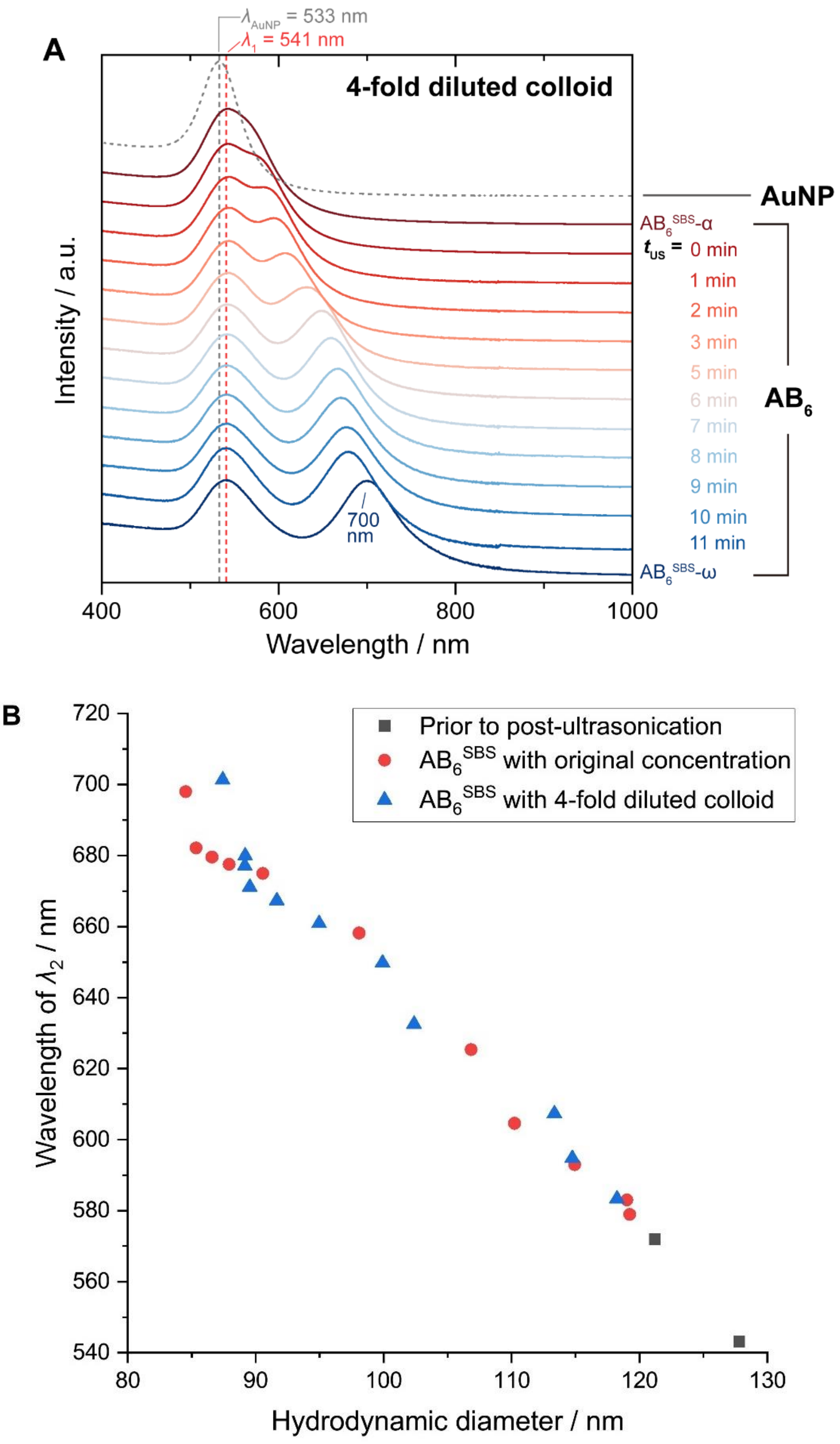

Figure S2. **(A)** UV-Vis spectra from an additional AB_6^{SBS} experiment series. Instead of using the original concentration as in Figure 4A–C in the main text, a four-fold diluted AB_6^{SBS}-*α* was used here. **(B)** Comparison of AB_6^{SBS} experiments using the original concentration and a four-fold diluted colloid.

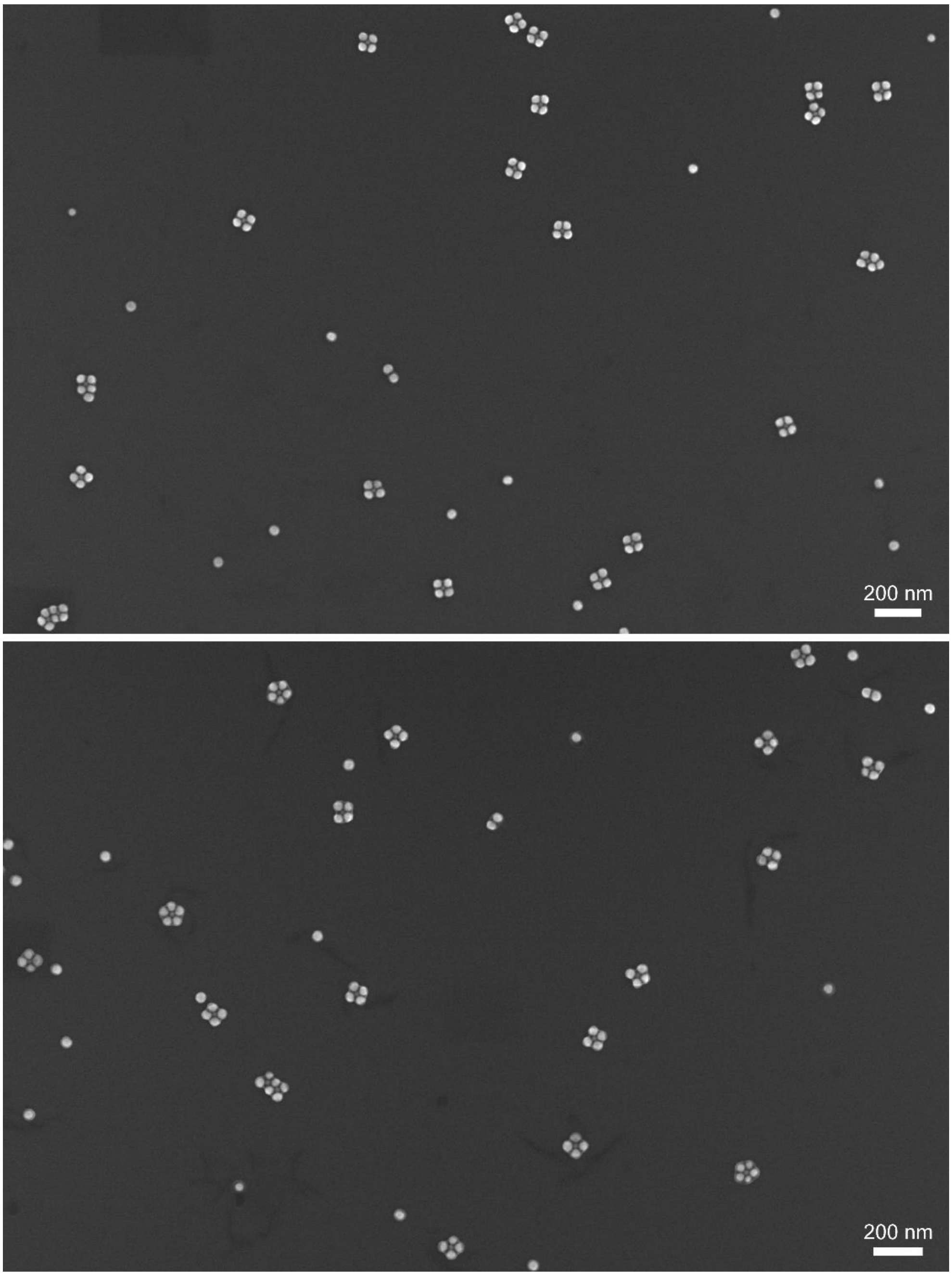

Figure S3. Additional SEM images of 2D AB_4 PMs resulting from the drop-casting of AB_4-α colloid onto a silicon substrate. PEG_{136}-grafted 22 nm A-NPs and P($St_{0.7}$-*r*-$HSt_{0.3}$)$_{420}$-grafted 34 nm B-NPs were used, respectively.

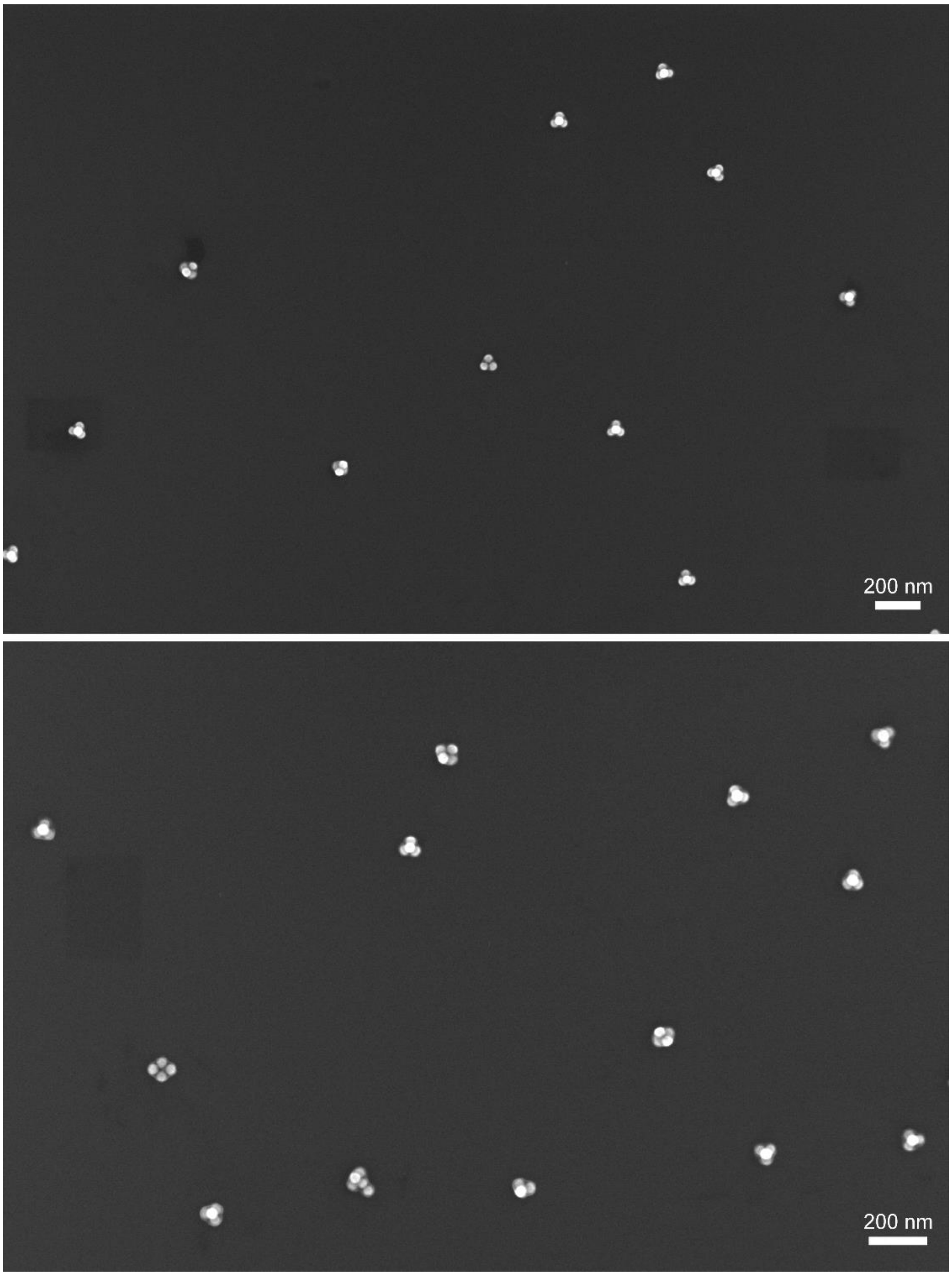

Figure S4. Additional SEM images of 3D AB_4 PMs resulting from the drop-casting of AB_4-ω colloid onto a silicon substrate. PEG_{136}-grafted 22 nm A-NPs and P($St_{0.7}$-*r*-$HSt_{0.3}$)$_{420}$-grafted 34 nm B-NPs were used, respectively.

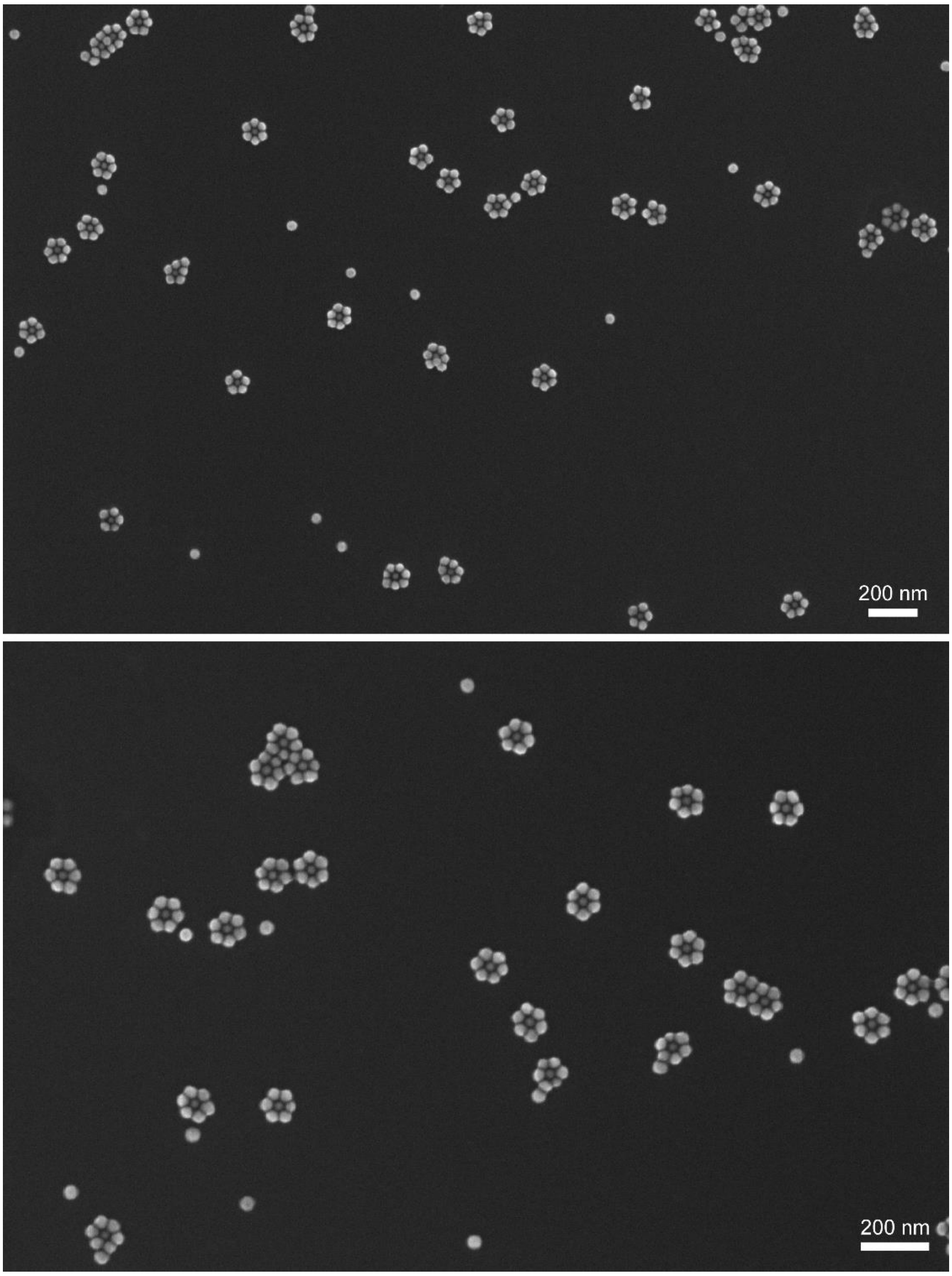

Figure S5. Additional SEM images of 2D AB_6 PMs resulting from the drop-casting of AB_6-α colloid onto a silicon substrate. PEG_{227}-grafted 28 nm A-NPs and $P(St_{0.7}$-r-$HSt_{0.3})_{420}$-grafted 34 nm B-NPs were used, respectively.

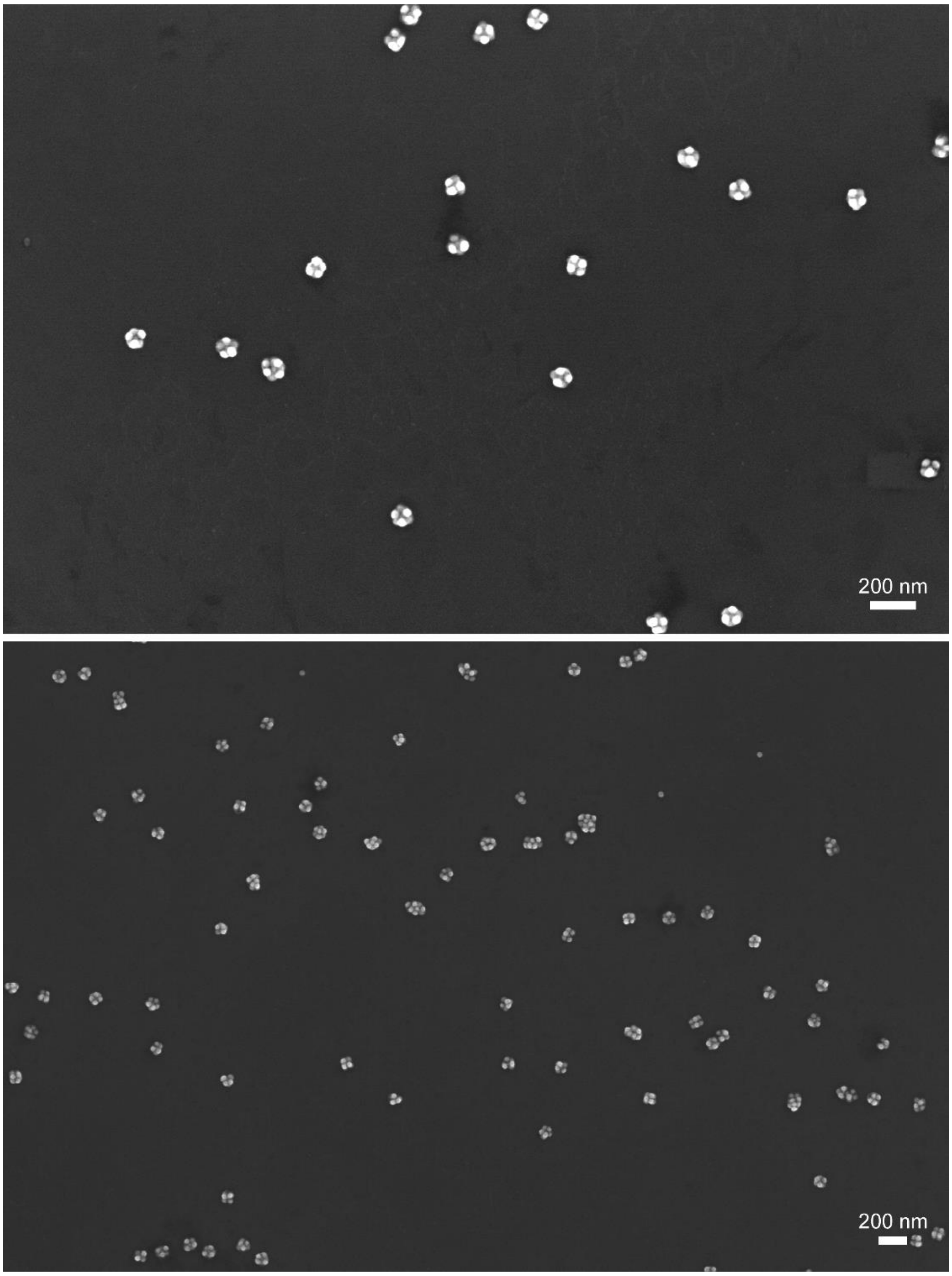

Figure S6. Additional SEM images of 3D AB_6 PMs resulting from the drop-casting of AB_6-*ω* colloid onto a silicon substrate. PEG_{227}-grafted 28 nm A-NPs and $P(St_{0.7}\text{-}r\text{-}HSt_{0.3})_{420}$-grafted 34 nm B-NPs were used, respectively.

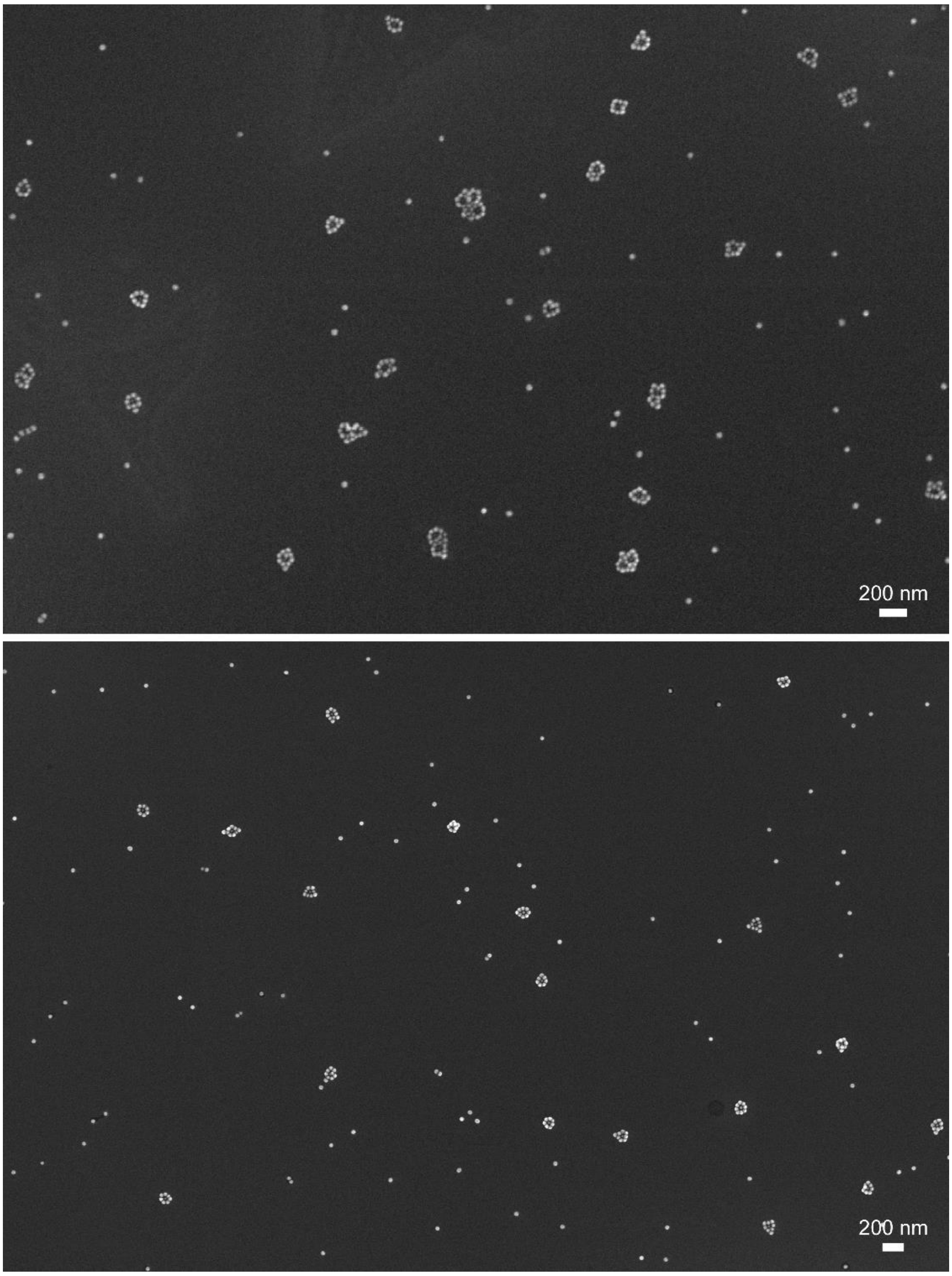

Figure S7. Additional SEM images of 2D AB_8 PMs resulting from the drop-casting of AB_8-α colloid onto a silicon substrate. PEG_{227}-grafted 28 nm A-NPs and P($St_{0.7}$-*r*-$HSt_{0.3}$)$_{420}$-grafted 34 nm B-NPs were used, respectively.

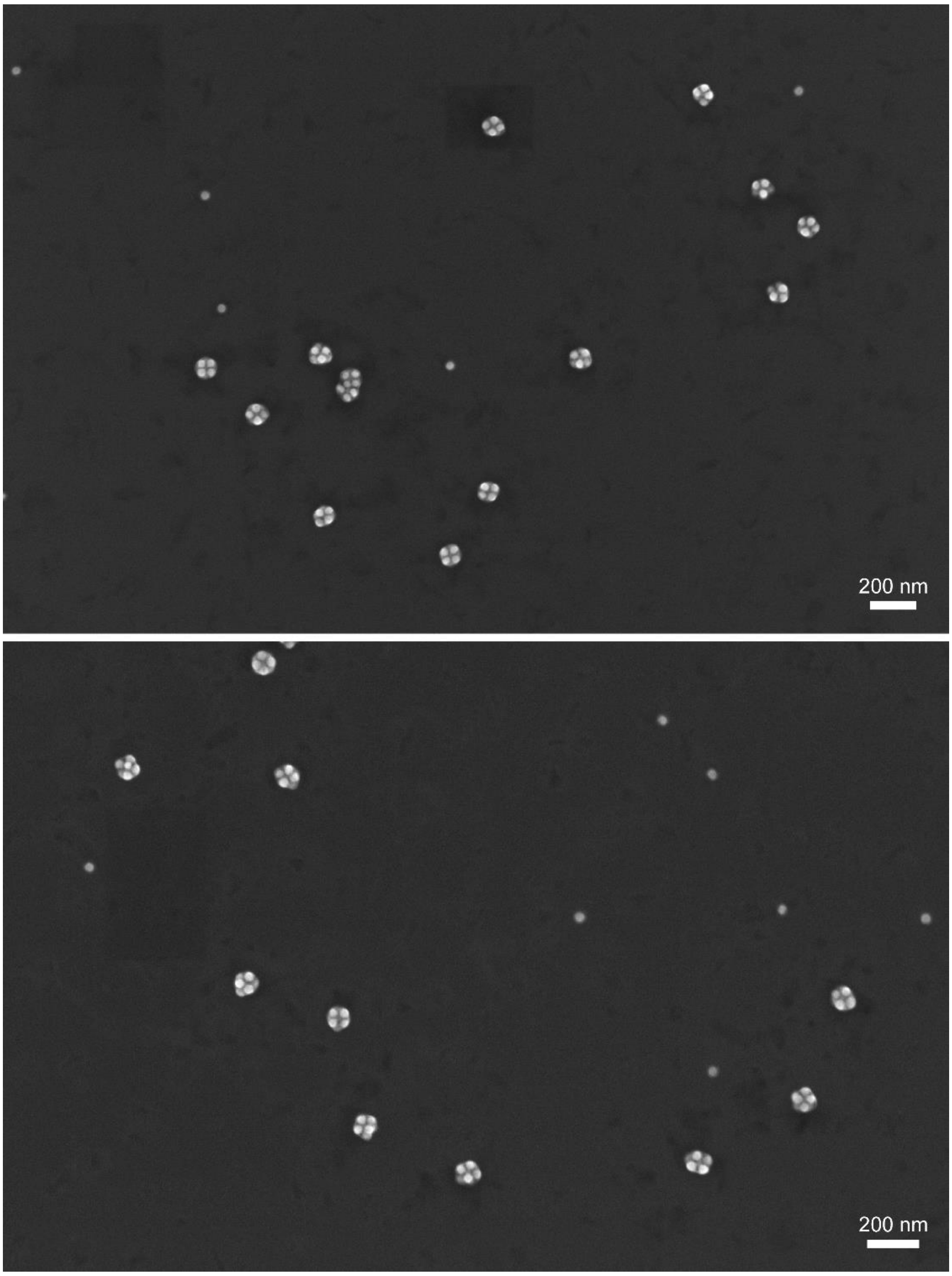

Figure S8. Additional SEM images of 3D AB_8 PMs resulting from the drop-casting of AB_8-ω colloid onto a silicon substrate. PEG_{227}-grafted 28 nm A-NPs and P($St_{0.7}$-*r*-$HSt_{0.3}$)$_{420}$-grafted 34 nm B-NPs were used, respectively.

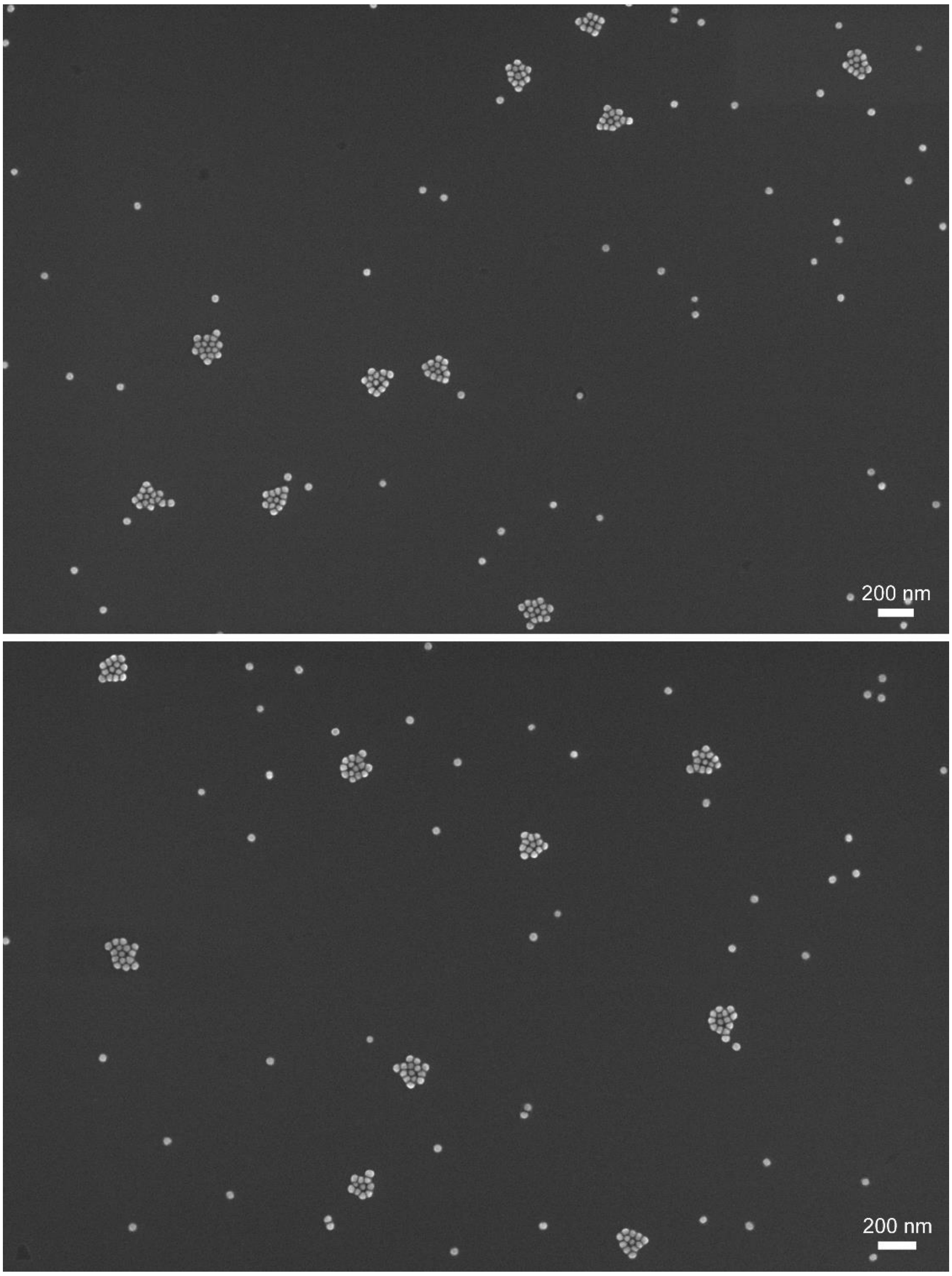

Figure S9. Additional SEM images of 2D AB_{12} PMs resulting from the drop-casting of AB_{12}-α colloid onto a silicon substrate. PEG_{454}-grafted 30 nm A-NPs and $P(St_{0.7}$-r-$HSt_{0.3})_{420}$-grafted 34 nm B-NPs were used, respectively.

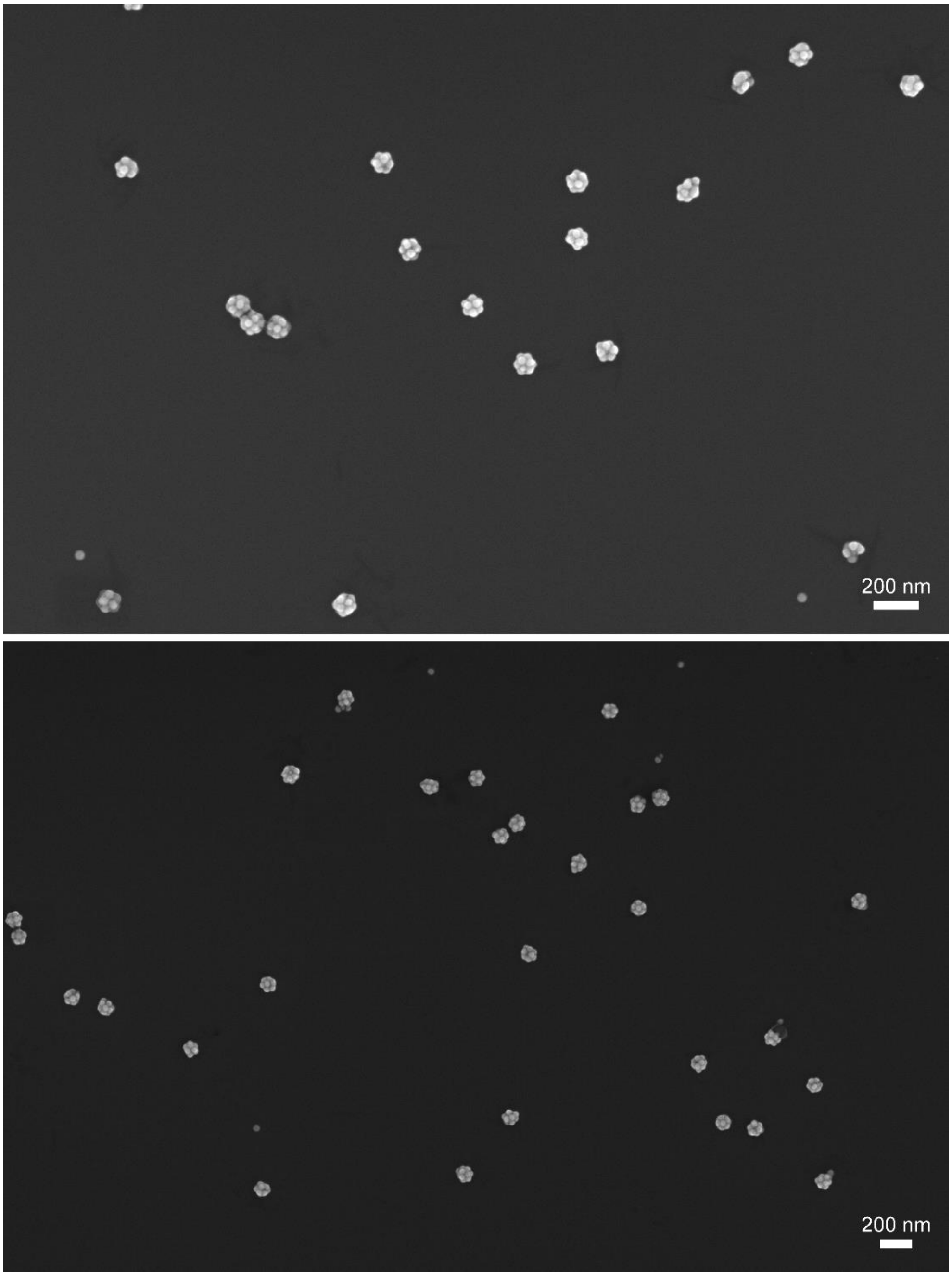

Figure S10. Additional SEM images of 3D AB_{12} PMs resulting from the drop-casting of AB_{12}-ω colloid onto a silicon substrate. PEG_{454}-grafted 30 nm A-NPs and $P(St_{0.7}$-*r*-$HSt_{0.3})_{420}$-grafted 34 nm B-NPs were used, respectively.

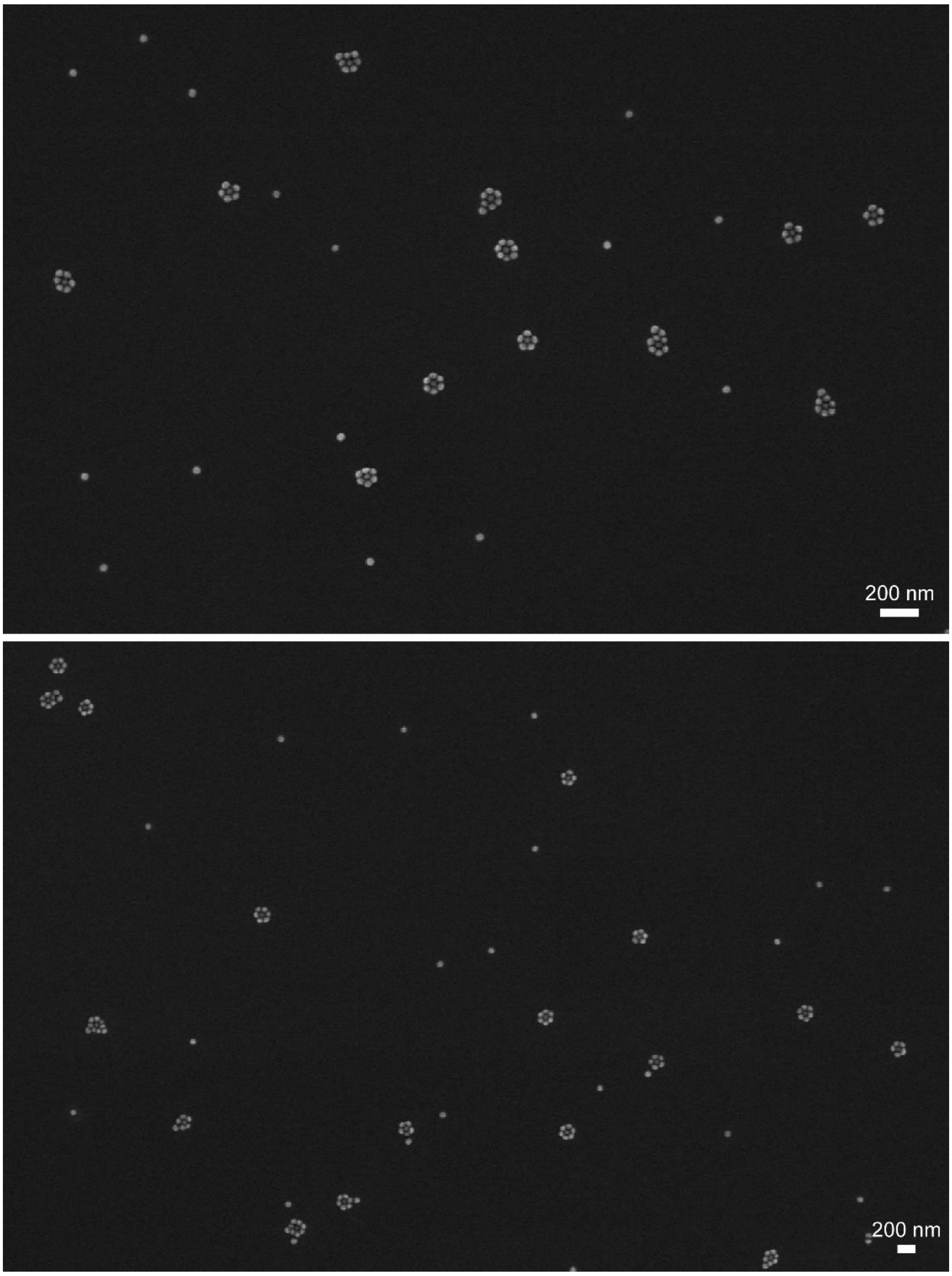

Figure S11. SEM images of 2D AB_6 PMs resulting from the drop-casting of AB_6^{SBS}-α colloid. PEG_{227}-grafted 34 nm A-NPs and $P(St_{0.7}$-r-$HSt_{0.3})_{420}$-grafted 34 nm B-NPs were used, respectively.

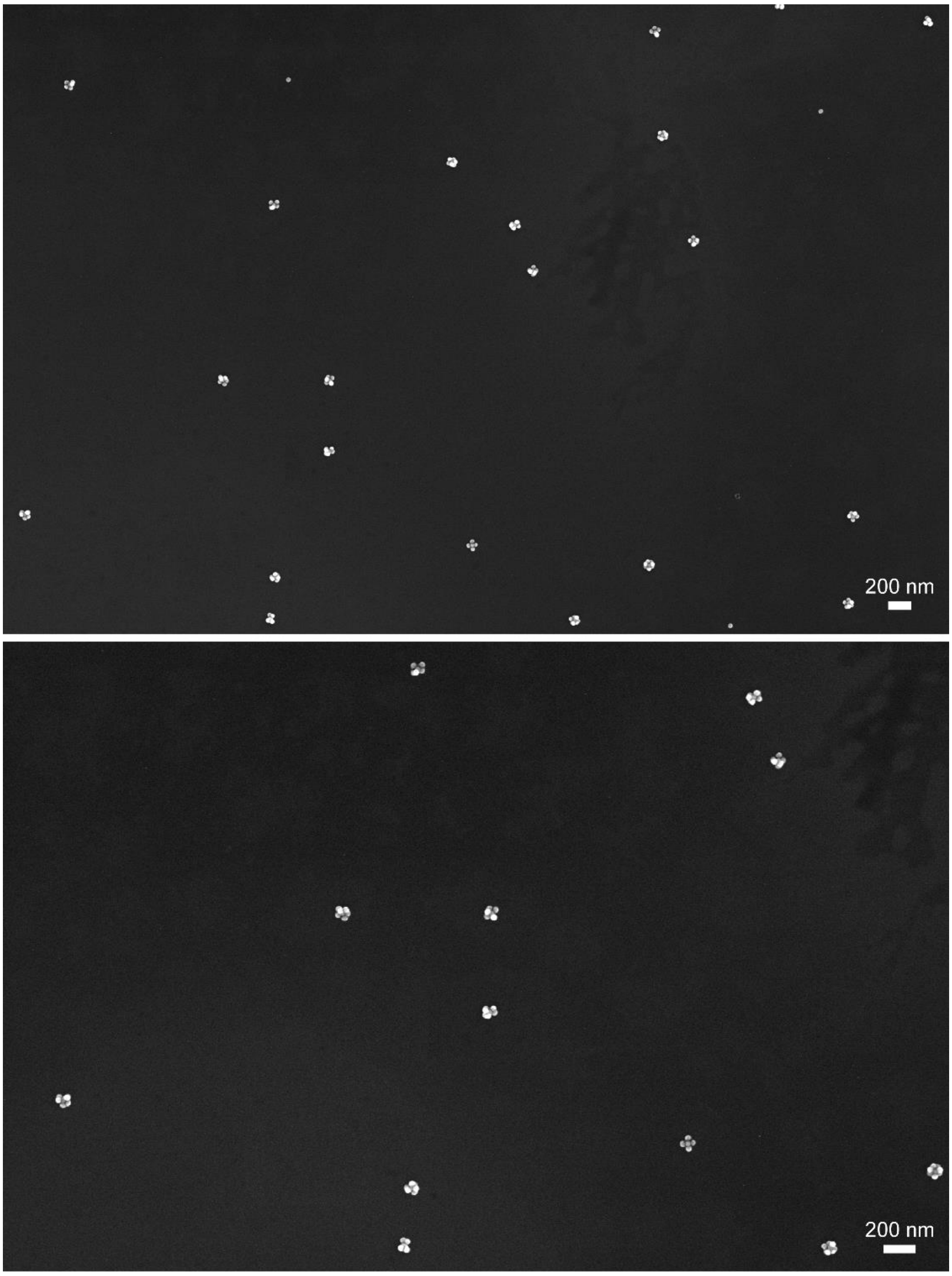

Figure S12. SEM images of AB_6 PMs resulting from the drop-casting of AB_6^{SBS}(t_{US} = 20 min) colloid. PEG_{227}-grafted 34 nm A-NPs and $P(St_{0.7}$-*r*-$HSt_{0.3})_{420}$-grafted 34 nm B-NPs were used, respectively.

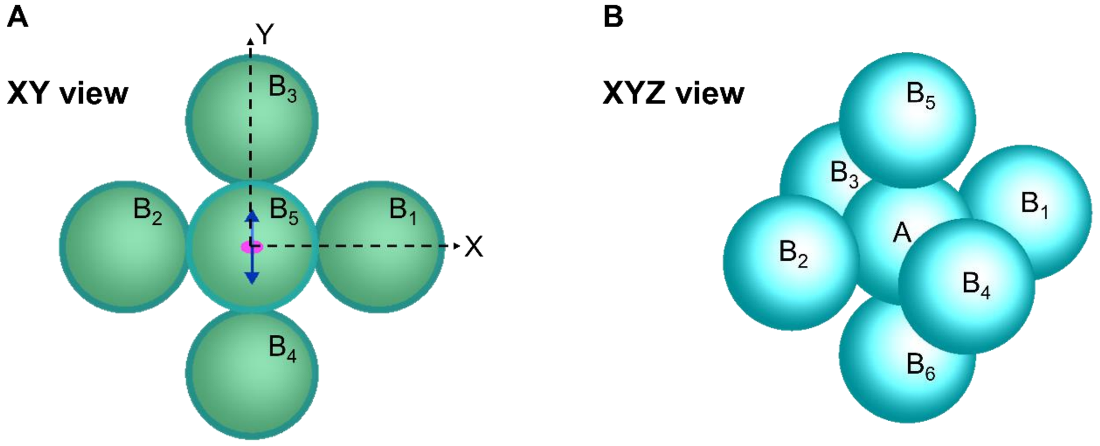

Figure S13. (A) Top view on the XY plane and **(B)** 3D view of the model used for FDTD-based simulations. The modeling parameters represent the 3D colloidal state of AB_6^{SBS} PMs: Spherical 34.3 nm AuNPs, covered with 2 nm polymer shells, are arranged in an octahedral geometry with A-NPs in the center. The $d_{A\text{-}B}$ is the edge-to-edge distance between the Au surfaces of the A- and B-NP.

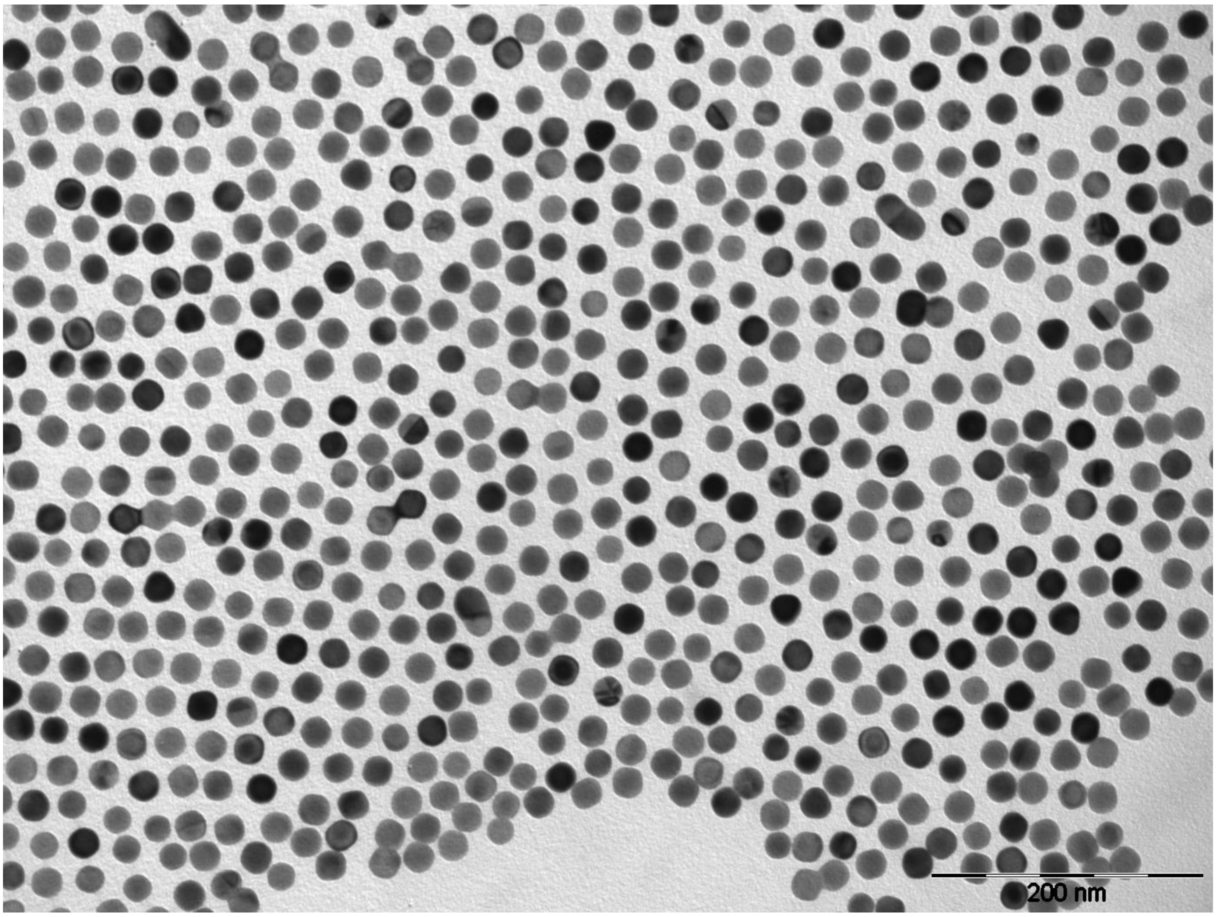

Figure S14. TEM image of PEG_{136}-grafted 22 nm A-NPs.

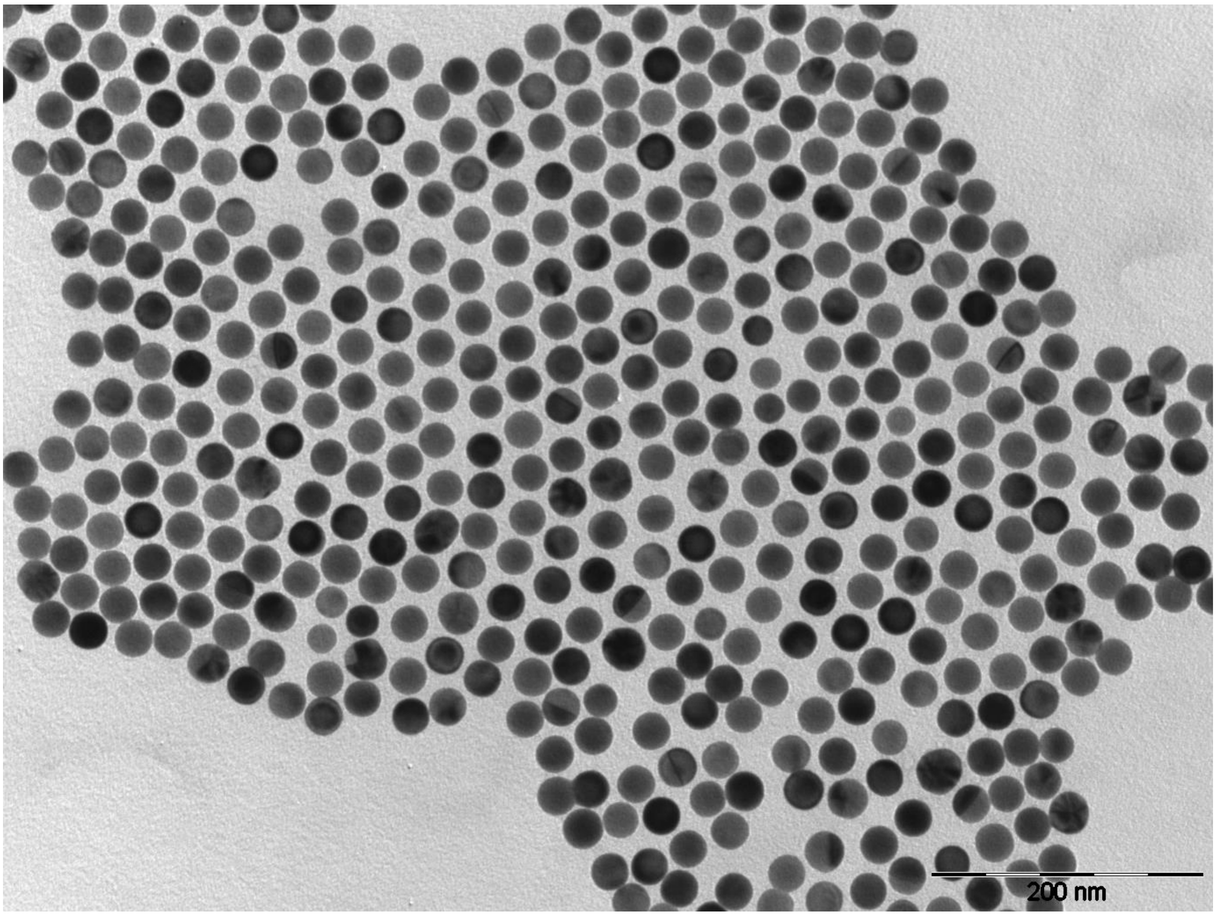

Figure S15. TEM image of PEG_{227}-grafted 28 nm A-NPs.

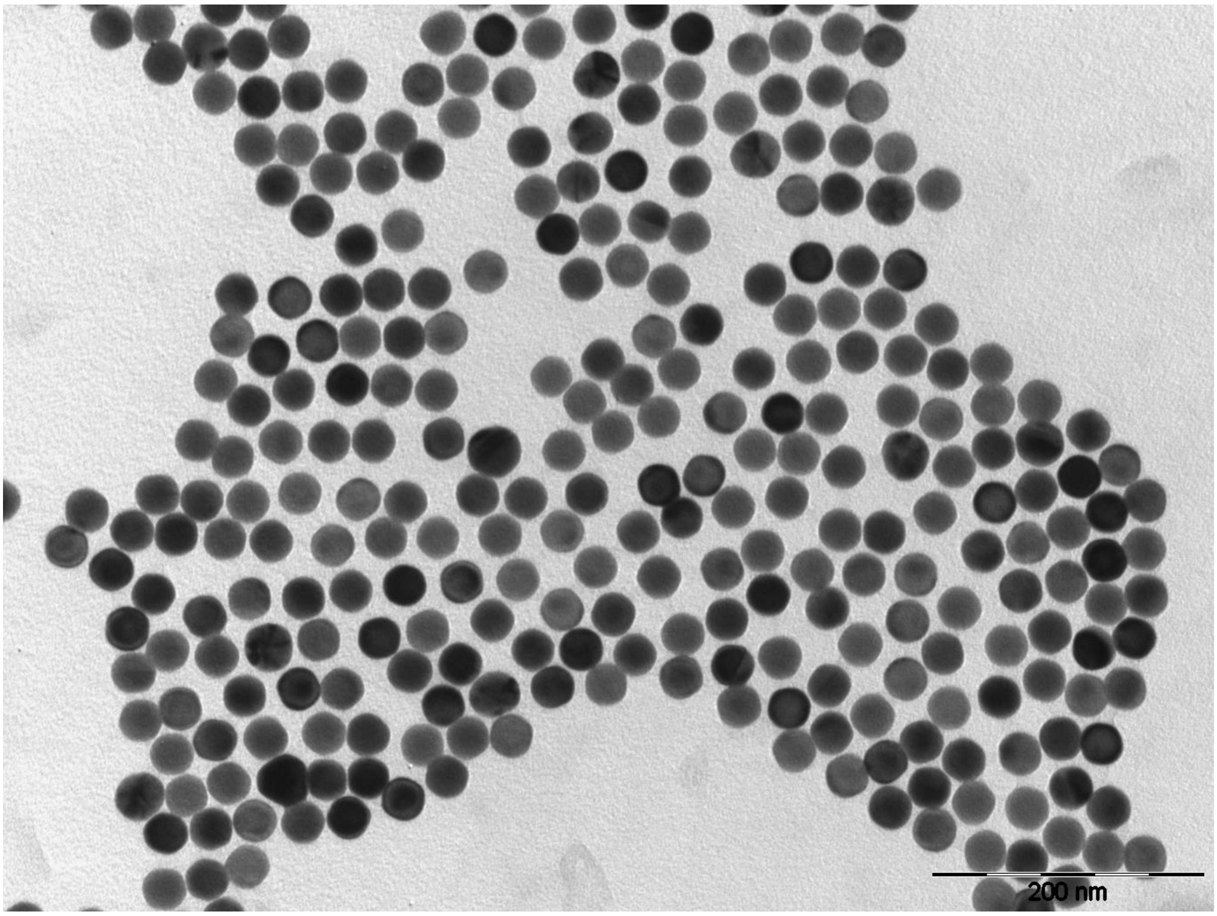

Figure S16. TEM image of PEG_{454}-grafted 30 nm A-NPs.

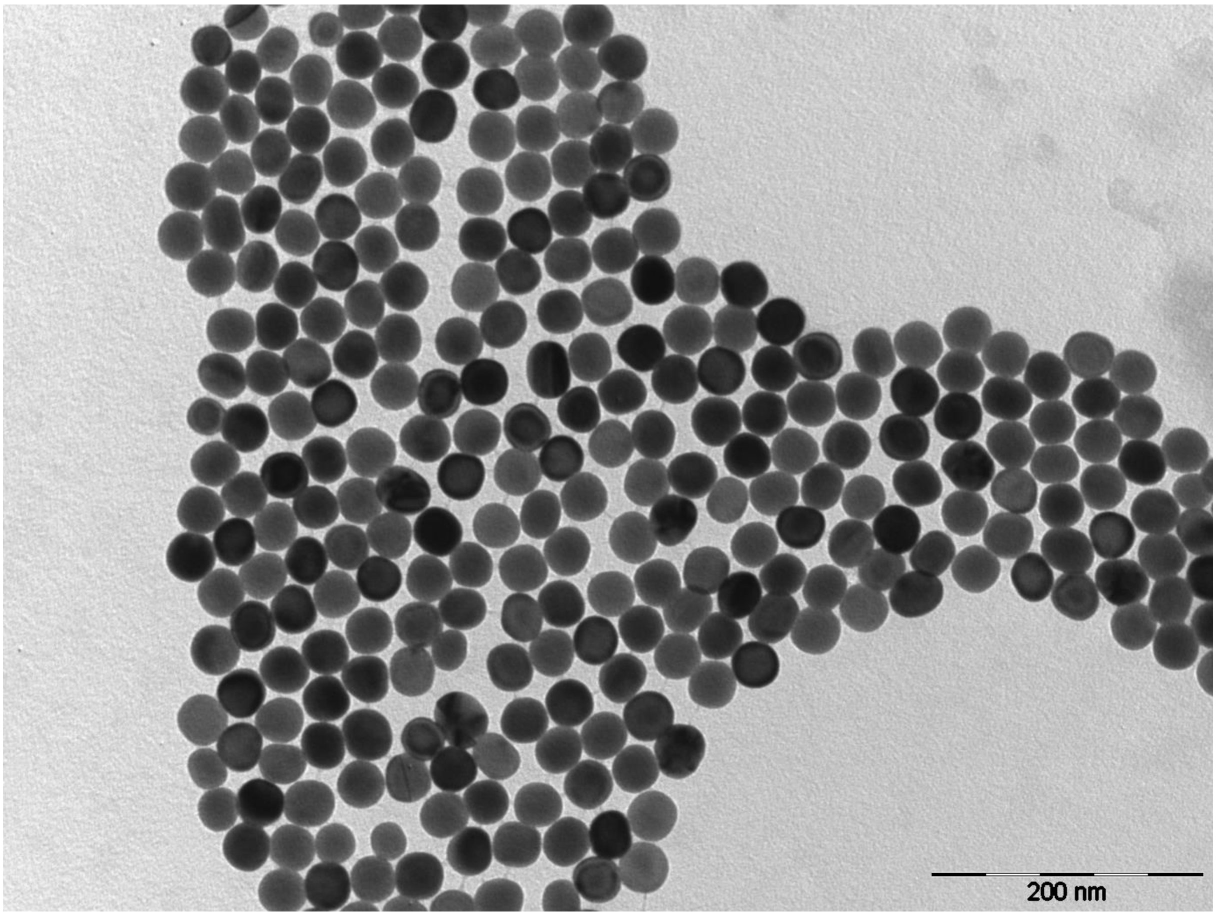

Figure S17. TEM image of PEG_{227}-grafted 34 nm A-NPs.

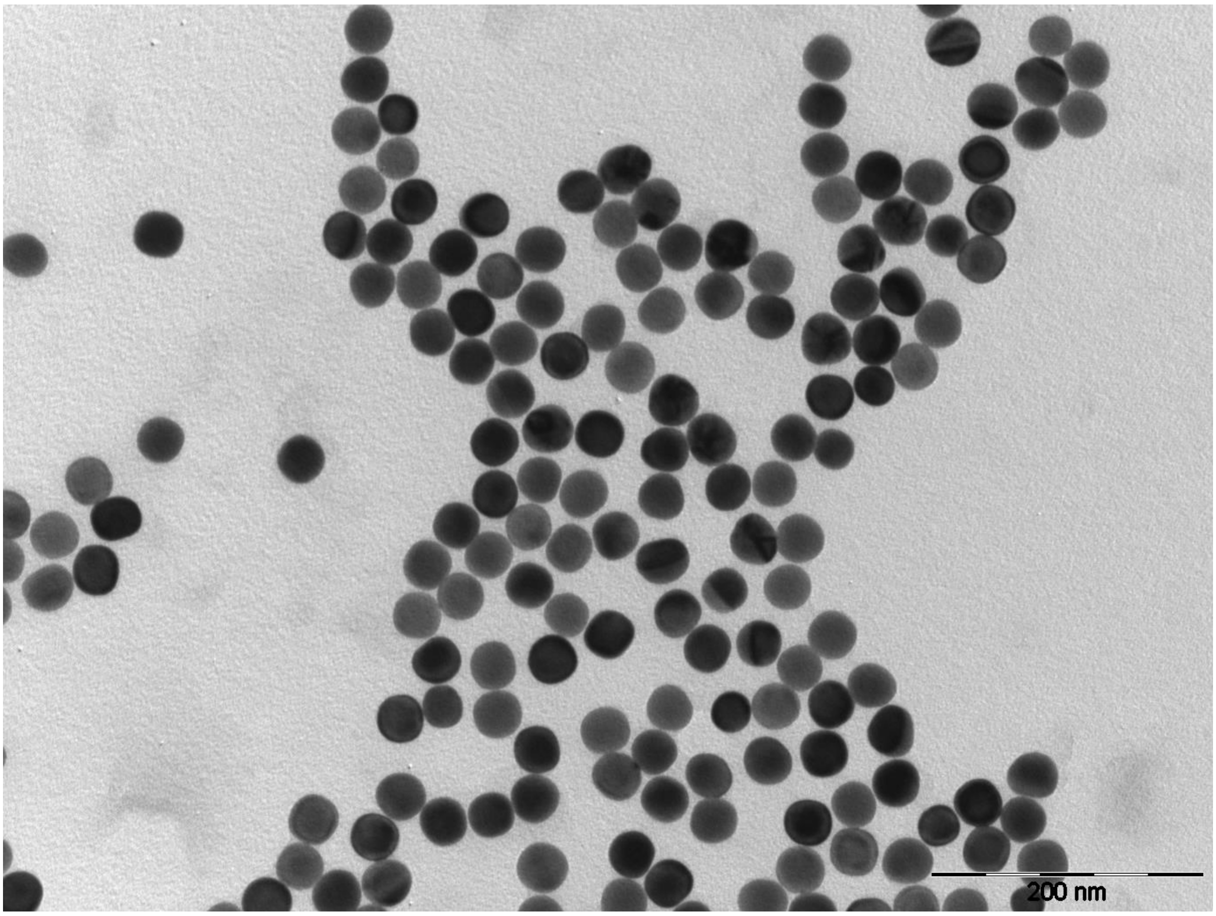

Figure S18. TEM image of P($St_{0.7}$-*r*-$HSt_{0.3}$)$_{420}$-grafted 34 nm B-NPs.

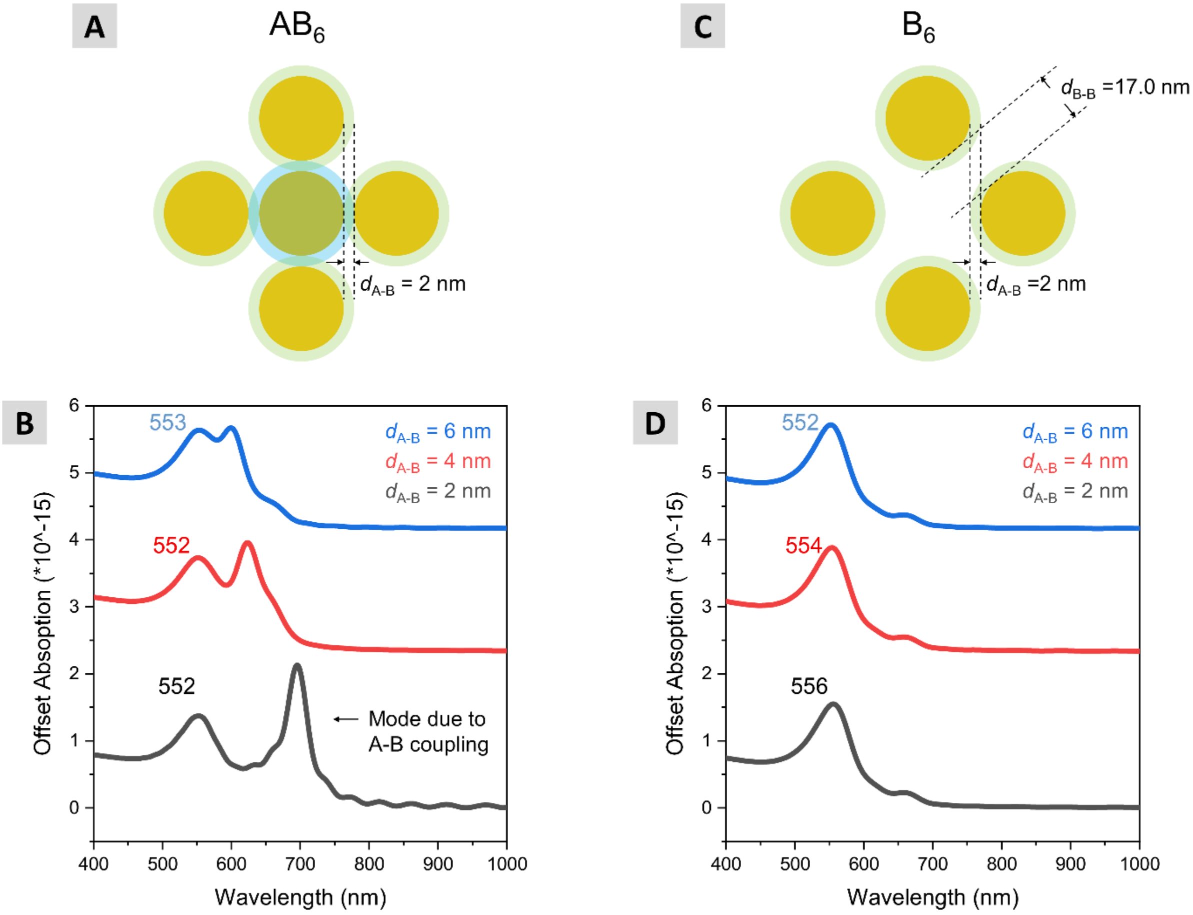

Figure S19. (A–B) Simulation model (cross-section in the XY plane) and the corresponding simulated absorption cross-section spectra of a 3D colloidal AB_6 PM with varied $d_{A\text{-}B}$ distances. For simplicity, all AuNPs are set to have a diameter of 34.3 nm and covered with 2 nm polymer shells. **(C–D)** Simulation model (cross-section in the XY plane) and simulated absorption cross-section spectra of AB_6 with removed central A-NP. The removal of A-NP results in the disappearance of the A-B coupled mode. No significant B-B coupling is observed, even in the closest interparticle configurations, as the B-B distance remains too large (>17 nm) to facilitate coupling events. Slight redshifting originates from the increase in the surrounding refractive index due to a higher packing fraction per unit volume.

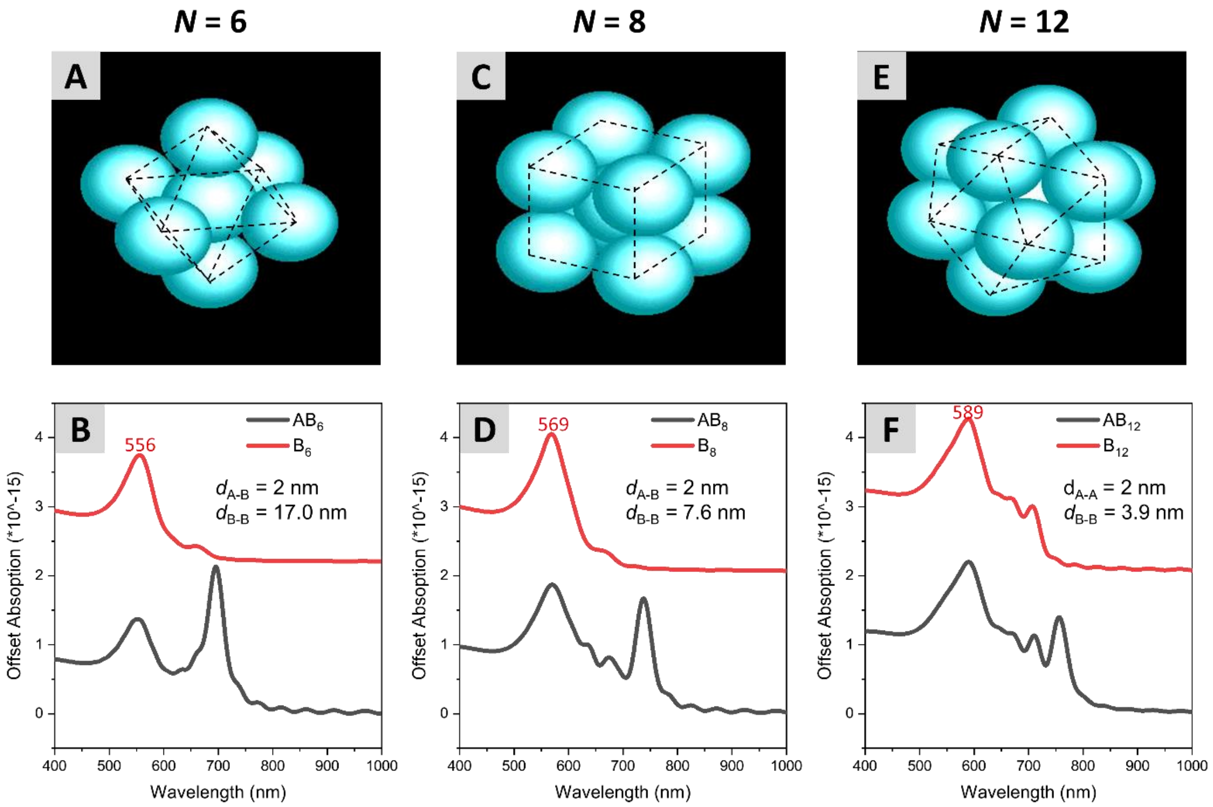

Figure S20. Comparison of simulation results for 3D colloidal AB_6 (A–B), AB_8 (C–D), and AB_{12} (E–F) PMs with $d_{A\text{-}B}$ = 2 nm. **(A, C, E)** The corresponding 3D models for colloidal AB_N PMs. For simplicity, all AuNPs are set with a diameter of 34.3 nm and covered with 2 nm polymer shells. **(B, D, F)** The simulated absorption spectra for AB_N structures versus B_N structures (with the central A-NP removed) illustrate the changing trends in plasmon hybridization as the coordination number increases. **(B)** In AB_6, no B-B coupling occurs due to the large B-B distance. The second peak in the AB_6 spectrum appears mainly due to A-B coupling. **(D, F)** As N increases to 8 and then to 12, B-B coupling begins to take effect due to the drastically decreased B-B distance, as evidenced by significant red-shifting and the emergence of further collective modes in the B_8 and B_{12} spectra. Both AB_8 and AB_{12} spectra exhibit hybridization between the collective mode from B_N and the central A-NPs, indicative of Fano resonance.

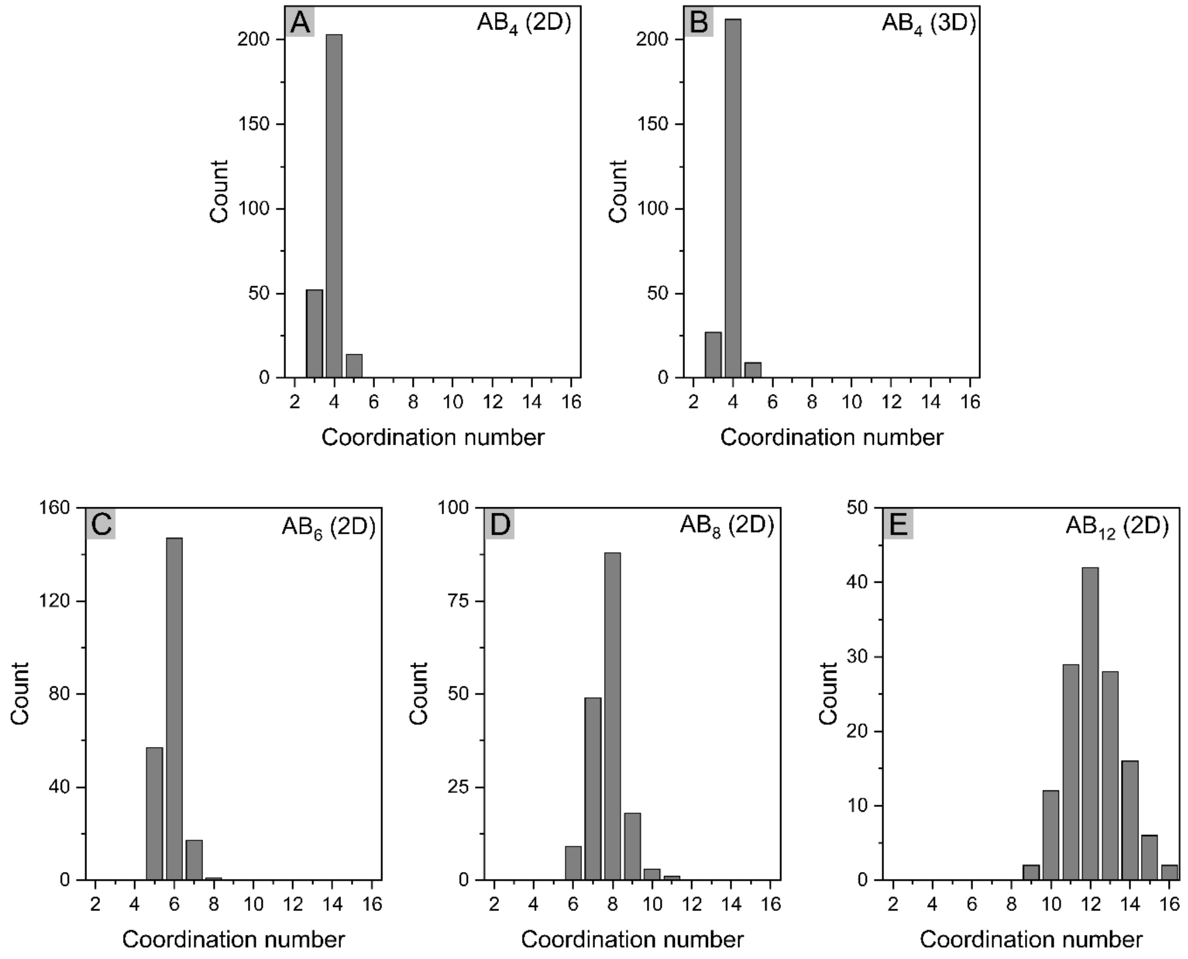

Figure S21. Histograms of N values for AB_4, AB_6, AB_8, and AB_{12} samples in 2D or 3D states. The evaluation is conducted by manually counting at least 137 clusters for each sample. **(A, C–E)** The histograms of N for 2D PM samples which correspond to the relative frequency diagrams in Figure 2C. For PMs with higher N values (AB_8 and AB_{12}), where it is challenging to distinguish A-NPs from B-NPs due to their similar sizes, the N value is calculated by counting the total number of NPs in each cluster and then reducing this number by one to account for the A-NP, assuming each PM contains only one A-NP. **(A–B)** The comparison of histograms between 2D ($N = 3.9 \pm 0.5$) and 3D ($N = 3.9 \pm 0.5$) states of AB_4 shows no significant changes, indicating no dissociation of NPs during the ultrasonication process. The statistical evaluation for 3D PMs with higher N is not included due to the difficulty of counting caused by the occlusion of upper AuNPs over the lower ones.

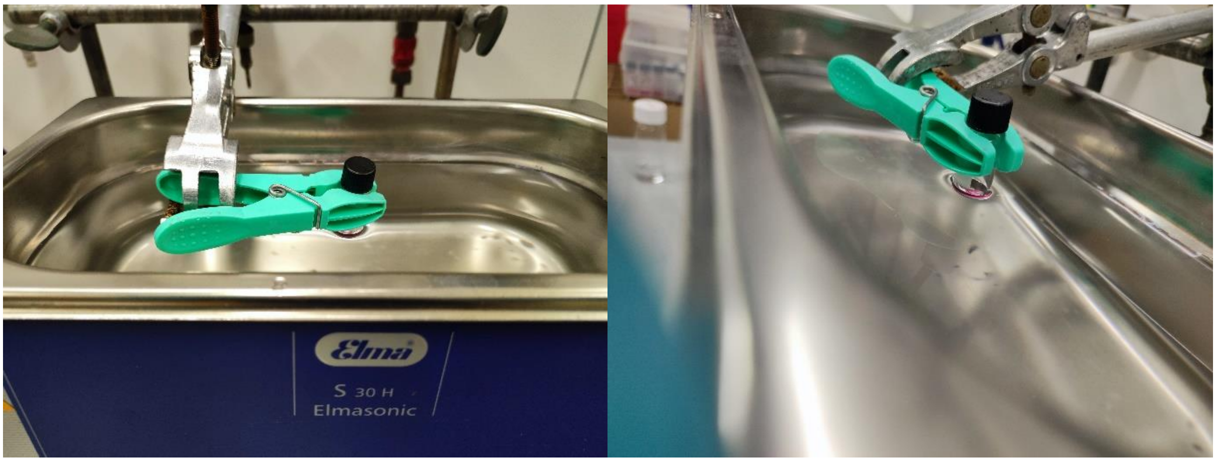

Figure S22. Photographs of the setup for the post-ultrasonication process.

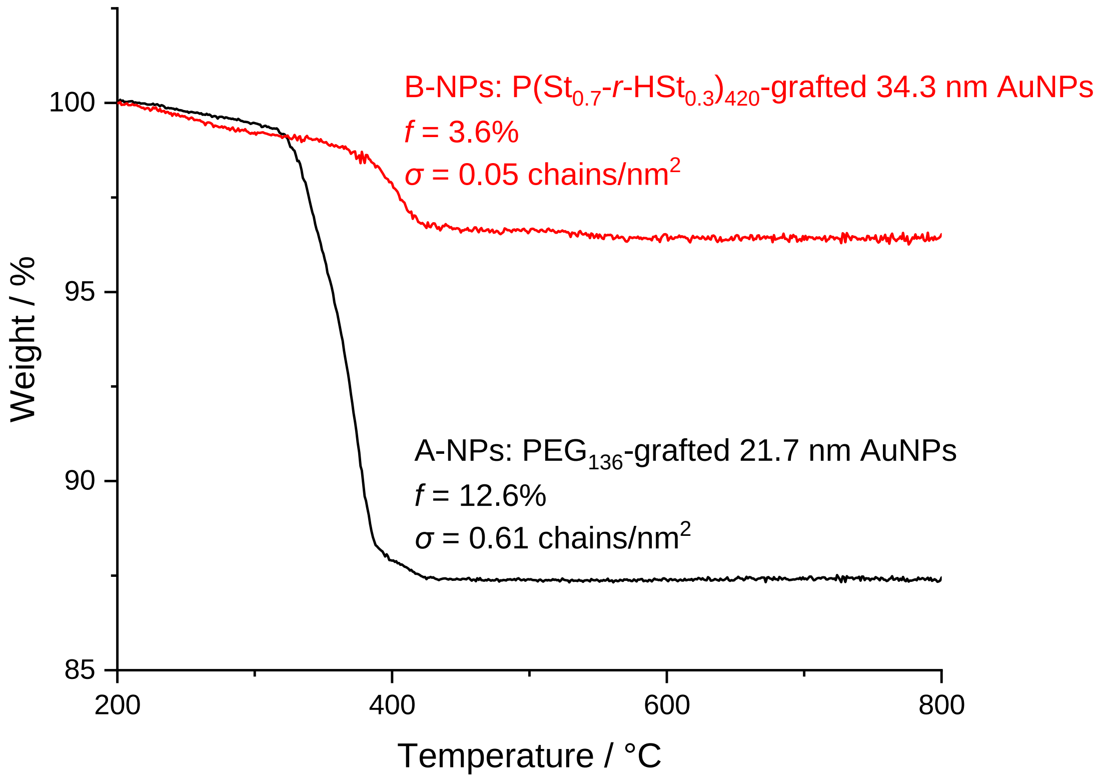

Figure S23. TGA curves of PEG_{136}-grafted 21.7 nm AuNPs (A-NPs) and $P(St_{0.7}$-*r*-$HSt_{0.3})_{420}$-grafted 34.3 nm AuNPs (B-NPs), respectively. The total weight loss (*f*) was obtained once the weight stabilized in the temperature region above 600°C. The capping density (σ) was estimated to be 0.61 chains/nm² for A-NPs and 0.05 chains/nm² for B-NPs using Equation S1. The high capping density of the NPs after 13 wash cycles indicates that there was no polymer detachment from the gold surface during the repeated centrifugation and redispersion processes, which included ultrasonication.

5. References

[1] P. A. Hassan, S. Rana, G. Verma, *Langmuir* **2015**, *31*, 3–12.

[2] H. N. Sólimo, A. C. Gomez Marigliano, *J. Solution Chem.* **1993**, *22*, 951–962.

[3] Y. Cai, P. Vana, *Angew. Chem. Int. Ed.* **2023**, *62*, e202309798.

[4] L. Scarabelli, A. Saánchez-Iglesias, J. Peérez-Juste, L. M. Liz-Marzaán, *J. Phys. Chem. Lett.* **2015**, *6*, 4270–4279.
